# Supplementary material for: Experiences of seeking healthcare across the border: lessons to inform upstream policies and system developments on cross-border health in East Africa
Source: BMJ Open. 2021 Dec 1;11(12):e045575. doi: 10.1136/bmjopen-2020-045575 (PMC8640642; doi:10.1136/bmjopen-2020-045575)
Supplement: Supplementary data [file bmjopen-2020-045575supp001.pdf]

Supplementary Table 1: Summary qualitative themes and sub-themes.

Reasons for crossing the border to seek health care		
Theme	Sub themes	Illustrative quote
****Physical accessibility of the health services	Nearest health facilities is on the opposite side of the border	*“The reason I went to Uganda, it is because the health facilities where we get our vaccination (RUBAYA, CYUMBA and KANIGA) are more distant, and the transport to reach there is expensive” - Border resident, Gatuna-Rwanda.*
	Having relatives and friends on the opposite side of the border	*“My relative live there, so I went there because they could take care of me after giving birth …. I just crossed because I knew my family would receive me”-Border resident, Gatuna-Rwanda.*
***Affordability of the health services	Health services more affordable across the border	*“It all started when I was coming from the hospital … I needed medication to recover, but, the medicines I required, were not covered under my health insurance. So a friend of mine recommend me to go to Uganda side”- Border resident, Gatuna-Rwanda.*
**Availability of the health services	Presence of needed services across the border	*“I took my grandchild because after visiting the hospitals here for three consecutive times and missing the vaccine, I was forced to travel to Uganda for the vaccine”- Border Resident, Busia-Kenya.*
	Formal referral by health workers at home	*“I started feeling labor pains where I visited the district hospital but got students who were attending to patients. I was bleeding and was advised to go to MASAFU (Uganda side) for delivery”- Border resident, Busia-Kenya.*
	Informal referrals by self, friends and other community members	*“…when I was sick, my employer she asked if I could have caught HIV. Therefore, she sent me to MAYENGO (Uganda side) for diagnosis, and I found that I had HIV”-Border resident, Busia-Kenya.*
*Acceptability of health services	Confidentiality of care sought across the border	*“When I discovered I was infected with HIV I was stigmatized and couldn’t stand going to pick drugs as I could meet very many people I know. I was forced to go to Uganda and gained courage when picking the drugs”-Border resident, Busia-Kenya.*
	Perceived better quality of care across the border	*“When I went to the Kenyan hospital they worked on me well and I came back safely. The good thing in Kenya is that any nurse can help you, she tells you where to start from, and she directs where to get the book and notes all things concerning the baby.”- Border resident, Busia-Uganda*
Experiences on how border resident communities navigated barriers during cross border health care access		
Theme	Sub themes	Illustrative quote
****Ability to physically cross into the neighbouring country	Presence of informal routes	*“Border officers send you back because all those services are available here….(in Rwanda). Actually, most people who need those services use shortcuts to cross. Furthermore, Ugandans who take HIV treatment here (in Rwanda) also pass through there (the informal route)” – Border resident, Gatuna-Rwanda*
	Official travel documents	*“No, whenever I visit there I am never asked for any document either when going or coming back”- Border Resident, Busia-Kenya.*
	Similarity in language	*“I was just treated well…It’s only the language barrier that made the whole process look tiresome”-Border Resident, Isebania-Kenya.*

****Ability to afford care across the border	Ability to meet direct cost of care	*"The cost was so much reasonable as compared to Kenyans side and we also used Kenyan currency which is stronger that the Uganda shillings"- Border Resident, Busia-Kenya,*
	The indirect costs of care	*"While on the way we encountered the police whom we bribed to proceed with our journey to Dabani Hospital. At the hospital we had a midwife who was attending to her by bringing her food and water to bath and we ended up paying her (the midwife)"-Border Resident, Busia-Kenya.*
