# Supplementary material for: Experiences of seeking healthcare across the border: lessons to inform upstream policies and system developments on cross-border health in East Africa
Source: BMJ Open. 2021 Dec 1;11(12):e045575. doi: 10.1136/bmjopen-2020-045575 (PMC8640642; doi:10.1136/bmjopen-2020-045575)
Supplement: Author's manuscript [file bmjopen-2020-045575.draft_revisions.pdf]

BMJ Open is committed to open peer review. As part of this commitment we make the peer review history of every article we publish publicly available.

When an article is published we post the peer reviewers' comments and the authors' responses online. We also post the versions of the paper that were used during peer review. These are the versions that the peer review comments apply to.

The versions of the paper that follow are the versions that were submitted during the peer review process. They are not the versions of record or the final published versions. They should not be cited or distributed as the published version of this manuscript.

BMJ Open is an open access journal and the full, final, typeset and author-corrected version of record of the manuscript is available on our site with no access controls, subscription charges or pay-per-view fees (http://bmjopen.bmj.com).

If you have any questions on BMJ Open's open peer review process please email info.bmjopen@bmj.com

BMJ Open

Experiences of seeking health care across the border: Lessons to inform upstream policies and system developments on cross border health in East Africa

Journal:	*BMJ Open*
Manuscript ID	bmjopen-2020-045575
Article Type:	Original research
Date Submitted by the Author:	05-Nov-2020
Complete List of Authors:	Ssengooba, Freddie; Makerere University, School of Public Health, Department of Health Policy Planning and Management; Makerere University School of Public Health, SPEED Project<br>Tuhebwe, Doreen; Makerere University School of Public Health, Department of Health Policy Planning and Management<br>Ssendagire, Steven; Makerere University School of Public Health, Department of Health Policy Planning and Management<br>Babirye , Susan ; Makerere University School of Public Health, Department of Health Policy Planning and Management<br>Akulume , Martha ; Makerere University School of Public Health, Department of Health Policy Planning and Management<br>Ssennyonjo, Aloysius; Makerere University School of Public Health, Department of Health Policy Planning and Management; Makerere University School of Public Health, SPEED Project<br>Rutaroh, Arthur; African Health Economics and Policy Association.<br>Mutesa, Leon; University of Rwanda, College of Medicine and Health Sciences<br>Nanagami, Mabel; Moi University College of Health Sciences, Health Policy and Management
Keywords:	Public health < INFECTIOUS DISEASES, PUBLIC HEALTH, Quality in health care < HEALTH SERVICES ADMINISTRATION & MANAGEMENT

SCHOLARONE™
Manuscripts

I, the Submitting Author has the right to grant and does grant on behalf of all authors of the Work (as defined in the below author licence), an exclusive licence and/or a non-exclusive licence for contributions from authors who are: i) UK Crown employees; ii) where BMJ has agreed a CC-BY licence shall apply, and/or iii) in accordance with the terms applicable for US Federal Government officers or employees acting as part of their official duties; on a worldwide, perpetual, irrevocable, royalty-free basis to BMJ Publishing Group Ltd ("BMJ") its licensees and where the relevant Journal is co-owned by BMJ to the co-owners of the Journal, to publish the Work in this journal and any other BMJ products and to exploit all rights, as set out in our licence.

The Submitting Author accepts and understands that any supply made under these terms is made by BMJ to the Submitting Author unless you are acting as an employee on behalf of your employer or a postgraduate student of an affiliated institution which is paying any applicable article publishing charge ("APC") for Open Access articles. Where the Submitting Author wishes to make the Work available on an Open Access basis (and intends to pay the relevant APC), the terms of reuse of such Open Access shall be governed by a Creative Commons licence – details of these licences and which Creative Commons licence will apply to this Work are set out in our licence referred to above.

Other than as permitted in any relevant BMJ Author's Self Archiving Policies, I confirm this Work has not been accepted for publication elsewhere, is not being considered for publication elsewhere and does not duplicate material already published. I confirm all authors consent to publication of this Work and authorise the granting of this licence.

For peer review only

Experiences of seeking health care across the border: Lessons to inform upstream policies and system developments on cross border health in East Africa

Authors: Freddie Ssengooba*[1,2], Doreen Tuhebwe[1], Steven Ssendagire[1], Susan Babirye[1], Martha Akulume[1], Aloysius Ssennyonjo[1,2], Arthur Rutaroh[3], Leon Mutesa[4], and Mabel Nangami[5]

[1]. Department of Health Policy Planning and Management, Makerere University School of Public Health, Kampala, Uganda
[2]. Makerere University School of Public Health, SPEED Project
[3]. African Health Economics and Policy Association, Kampala, Uganda
[4]. University of Rwanda, College of Medicine and Health
Sciences Kigali, Rwanda
[5]. Department of Health Policy and Management, Moi University College of health Sciences, Eldoret, Kenya

*Corresponding author, sengooba@musph.ac.ug or freddie.ssengooba@gmail.com +256772509316

Key words: cross border, border residents, health access, universal health coverage, East Africa

Word count: 5369 (abstract, strengths, limitations, introduction, methods, results, discussion and conclusion)

Abstract

Introduction: For Universal Health Coverage goals to be advanced, there is need to target communities along the border who represent the far fang areas of the health system with multiple barriers to health care access – especially if from the other country.

Objectives: This study explored the experiences of border residents in East Africa.

Participants: A cross-sectional survey was conducted among 279 household adults among resident communities along selected national border sites for the countries in the East African Community.

Setting: Access to HIV treatment, emergency obstetric care and childhood immunization services from the opposite side of the border for a period of 5 years prior to this study was explored. We applied the health access framework and an appreciative inquiry approach that explored factors that enabled access to these three services.

For peer review only - http://bmjopen.bmj.com/site/about/guidelines.xhtml

Measures: Exploratory Factor Analysis and linear regression were used for quantitative data, while deductive content analysis was done for the qualitative data – i.e. respondent's experiences of navigating health access barriers.

Results: Nearly one third (77/279) of the respondents had accessed care from the opposite side of the border. The majority (88.9%; 234/279) accessed care from public health facilities. From the linear regression models, the main predictor for ease of access for health care were ''ease of border crossing" (Regression Coefficient (RegCoef) 0.381); "services being free" (RegCoef 0.478); "services and medicines availability" (RegCoef 0.274) and "acceptable quality of services" (RegCoef 0.364). The key facilitators for successful navigation of access barriers were related to the presence of informal routes, speaking a similar language and the ability to pay for the services received.

Conclusion: Communities resident near national borders were able to cross borders to seek health care. There is need for a policy environment to enable East Africa invest better and realize synergies for the border resident communities.

Strengths and limitations of this study

- The study involved the use of both qualitative and quantitative data which strengthened the triangulation of results
- Having multi country study sites in the East Africa Community strengthened the study by capturing diverse experiences that can inform cross border cooperation initiatives.
- This paper only presents the views of respondents that successfully navigated access barriers.
- Our study involved communities adjacent to the official cross points. We did not include communities along the numerous informal cross points which may have different contexts and experiences.

For peer review only - http://bmjopen.bmj.com/site/about/guidelines.xhtml

Introduction

Border resident communities are the population settlements, rural or urban, in close proximity to the borders[1]. This term usually applies to international borders and the border resident communities of the two neighbouring countries are usually economically and socially interdependent [2] and have more in common with their sister communities across the border than with communities on their own side of the border. Residents of both countries cross the border for a variety of reasons, including; work, shopping, health care, visits with friends or relatives, and others [3].

Increasingly, border resident communities cross to access care in each other's country [4]. This is in partly due to challenges of accessing the needed care in the home country where the nearest health facility may be far away into their country and the border residents are located at the periphery with physical and economic access barriers. Cross-border health care can also enhance individual choice, by providing added option to obtain health care which may either exhibit a better quality or be available at a lower cost in comparison with their national provision system [5,6]. A study by Su and colleagues showed that one of the most significant predictors of health care utilization in Mexico by US citizens were lack of US health insurance coverage and dissatisfaction with the quality of care in the US[7]. Scholars have also proposed several frameworks that can be used to understand facilitators and barriers to health access[8-10].

However, access to care in the neighbouring country may not always be easy given the several barriers experienced along the pathway to care[2]. Some of the barriers to physical access may include challenges of poor road network and terrain and lack of identity documentation[11]. It could be an issue of financial barriers like high cost of care across the border despite of physical proximity, need for foreign currency and un-transferability of health insurance policy to a neighbouring country[5]. Other hindrances include; lack of quality health care infrastructure, health care workforce shortages and language barriers that pose acceptability challenges [3,12]. These barriers to access and utilization are often times rooted in the differences within legal and institutional frameworks that confer entitlement to health care between countries as well as creating differences in health care protocols [13].

Because, the nearest health facility or health services needed may be across the border, individuals and communities usually navigate these barriers in order to access care in the neighbouring country [14] though not institutionally recognised or planned for by the country receiving the clients. In the context of the East African Community (EAC), member states

For peer review only - http://bmjopen.bmj.com/site/about/guidelines.xhtml

(Uganda, Kenya, Tanzania, Rwanda, Burundi and South Sudan) have a progressive plan to increase the interaction and freedom of movements of commercial goods and people.

Literature has shown several mechanisms that border residents use to navigate health access barriers. A few of the strategies include using social and kinship networks which facilitate cross-border exchange of information about where care is available and where quality can be accessed [6,11]. This literature also shows that people take advantage of the cultural context and the ethno-linguistic similarities to navigate barriers such as ineligibility or special preconditions for noncitizens to health care services[15]. Because some of the border residents live as one community in the two opposite countries, there are usually many informal crossings through non-official crossing points for trade and inherently promote cross border health access.

Border communities, regardless of their size, are often regarded as peripheral in terms of social programs but paradoxically have high priority in terms of national security and in allocation of citizenry privileges[16]. Of recent, border communities have also become of importance for public health surveillance due to threats like multi-drug resistant TB[17], Ebola outbreak experience in West Africa[18] and the novel corona virus disease (COVID-19). These threats can and have traversed borders as a result of inflows and outflows of people and goods across borders. The disease surveillance objectives have in some places forced health systems across the borders to cooperate in the control of disease outbreaks[19]. It is not clear how this cooperation is extending to support day-to-day health care needs of communities along the borders.

Leaving no one behind is the mantra for Universal Health Coverage (UHC) and the Sustainable Development Goals (SDGs)[20]. Communities like those residing along the EAC borders must be targeted because they represent the far fang areas of the health system with multiple barriers to health care access. Beside distance from cities and towns, they also face administrative barriers arising from citizenship and movement to reach functional services – especially if from the other country. If not well articulated in policies and arrangements of health service provision, populations along the borders may remain vulnerable to poor access to health care and at risk of being left behind. This study set out to document experiences and feasible strategies to advance access and UHC goals especially for communities residing along the EAC borders. This study addressed two issues; 1) the health access reasons (physical, financial, availability and acceptability dimensions) why border residents cross or fail to cross to seek

For peer review only - http://bmjopen.bmj.com/site/about/guidelines.xhtml

health care across the border and 2) how they navigate institutional health system barriers and challenges of seeking health care across the border in East Africa.

Methods

Study setting and sites

This study was conducted among border resident communities in East Africa. We defined a border resident community as that community within 5-10 kilometers on either side of a designated international border crossing point, depending on whether the community is clustered at the exact crossing point or along the main highway that transits through the border crossing point. Four study sites were selected. These are; 1) Katuna-Uganda/Gatuna-Rwanda, 2) Rusomo-Rwanda/Lusomo-Tanzania, 3) Busia-Uganda/ Busia-Kenya and 4) Isebania-Kenya/ Sirare-Tanzania. The physical location of the study sights is shown in figure 1.

Study design and respondent population

A cross-sectional survey design was used. We collected data from a total 279 household adults who (or whose household members) had ever accessed HIV treatment refill, emergency obstetric care and childhood immunization services from the opposite side of the border within 5 years prior to this study. The three case conditions (HIV treatment refill, delivery and immunization) represent services aimed at chronic care, emergency care and prevention services respectively.

Data collection tool

An interviewer administered semi-structured questionnaire was used to survey the respondents. Questionnaire development was informed by the health access framework [8]. The questionnaire was divided into four sub-sections corresponding to four adapted dimensions (physical (geographical), financial (affordability), availability/accommodation and acceptability/appropriateness) of the health access framework. The geographical access, financial access, availability/accommodation and acceptability/appropriateness sub-sections of the questionnaire were operationalized by 15, 10, 16 and 5 Likert-scale (scale of 0-4) items respectively to assess the extent to which they agree (or disagree) that each of the items in the questionnaire facilitated their access to health care from the opposite side of the border. Each sub-section also had an overall Likert-scale item to assess overall ease with which that particular access dimension facilitated health care access.

For peer review only - http://bmjopen.bmj.com/site/about/guidelines.xhtml

The questionnaire had one open-ended question that asked each of the study respondents to give a narration of their most vivid experiences[21] seeking health care across the border. During the narration, we probed for factors that enabled navigation of barriers for example: cost of care, crossing requirements and quality of care. The narrations were audio recorded with consent of the respondents and transcribed verbatim. The questionnaire was validated through several rounds of iterations between the principal investigators and the 3 co-investigators of the study. In additional the tools were translated into the local language of the respective data collection site. The languages used included: Swahili (Kenya), Kinyarwanda (Rwanda), Rukiga (Uganda-Katuna border) and Lusoga (Uganda-Busia border).

Data collection procedure

At each study site, 75 survey respondents were targeted for survey and were divided equally across either side of the border for the three case conditions under study. To identify potential survey respondents, we asked the in-charges of the major catchment health facility on each of the study site to invite household adults in the health facility catchment area for a community dialogue. During the dialogue, the household adults (or any of their household members) who had ever accessed care from the opposite side of the border in the past five years were identified and categorized by case condition for which health care was accessed from the opposite side of the border. The required number of respondents was randomly sampled, consented and interviewed. The interviews were collected by a team of experienced and well-trained young researchers on the study team.

Data management and analysis

Filled questionnaires were checked for completeness, assigned a unique identifier and entered in *Epi Data*. The summary data set was then exported to *SPSS Version 23* for analysis. In preliminary analysis, we determined the reliability of the items in each of the 4 sub-sections of the questionnaire separately using the Cronbach's alpha reliability coefficient

Next, we conducted dimension reduction using Exploratory Factor Analysis (EFA) in *SPSS version 23* to generate fewer and meaningful (latent) variables with eigenvalues of 1 and above. To retain variables that clearly discriminate between the generated latent variables, the minimum cut off for item loading was set at 0.7.

Finally, a regression was performed to determine the independent association of each of the generated latent variables to the "perceived ease of access to health care" on the opposite side of the border. The strength and the statistical significance of the independent association of

For peer review only - http://bmjopen.bmj.com/site/about/guidelines.xhtml

each of the latent variables with the ease of cross-border health access was determined by their beta co-efficients, Confidence Intervals (CI) and critical P values. Results were stratified by disease condition and by dimension of the health access framework.

For the qualitative data, the recordings and all handwritten notes were kept in a secure location at the study secretariat office of the partnering universities that led the data collection. No identifying information of any of the specific respondents was included in the transcription and analysis reports. Qualitative data were exported to *Atlas TI version 8* for analysis. A conventional deductive content analysis approach was used [22].From the text, codes were derived then sorted into categories then grouped together to inform emerging overarching themes. The analysis took into considerations any relevant comparisons in the different study sites, country where health care was sought, gender and case/ disease condition. Relevant qualitative quotes from respondents were presented in the results sections to support the key results under the respective themes and categories.

The study was approved by the institutional review boards of the participating universities and the national research regulation councils in Uganda, Kenya, Tanzania and Rwanda were study activities were implemented. The community survey was not done in Tanzania due to unanticipated changes in the study team there and logistical challenges related to travel. All study respondents provided informed consent prior to participating in the study.

Patient and public involvement

Prior to the data collection field work, the study team conducted a study pre-visit meeting to all the study sites and we engaged local leaders, health officials and border managers in order to introduce the study objectives and seek their input in the data collection approaches. Through this collaboration with the local leadership, the team worked with some of the community members as field guides during the data collection. The study team also disseminated the study finding at all the border sites involved in the study in order for them to have an opportunity to validate the findings

Results

Respondent characteristics

A total of 279 households were surveyed. The majority of respondents were female (n=251, 90%), and married (n=213, 76.3%). Only 4 percent (n=11) were in formal employment. The

For peer review only - http://bmjopen.bmj.com/site/about/guidelines.xhtml

majority had relatives across the border in the neighboring country 75% (n=207). The detailed characteristics of the respondents are summarized in table 1.

Characteristics of individuals who accessed health-care from the opposite side of the border

Majority of the people who had accessed health care from the opposite side of the border were female (n=205, 73.5%), 27.6% (n=77) of whom had sought care more than a year ago. Majority accessed care from public health facilities (n=234, 88.9%) while 104 (37.3%) had sought immunisation services. The details are summarized in table 2.

Factors that support successful access to health care from the opposite side of the border

a) Reliability analysis of the survey questionnaire

The respondents were surveyed on four (4) dimensions of cross border health-care access. The reliability analysis of the survey questionnaire, reported as Cronbach's alpha showed the following results. Physical; 0.647, Affordability; 0.215, Availability and Accommodation; 0.783, Acceptability and Appropriateness; 0.645

Overall ease of cross border access to healthcare

The Likert -scale median scores ranged from 3-4. Overall, all access dimensions were important factors in successful cross border health access. For different case or disease conditions however, different access dimensions had different levels of contribution to successful cross border health access. For respondents who accessed emergency delivery services across the border, availability and accommodation dimension contributed more to their successful cross border health access. For respondents who accessed HIV treatment services, affordability contributed more. The details of these findings are summarized in figure 1.

The most significant factors that facilitate successful access to health-care from the opposite side of the border

We selected latent variables if they had eigenvalues exceeding 1.0, verifying the percent variance explained, cumulative percent of variance explained, and their scree plots[23]. Principle component analysis with varimax rotation generated latent variables for all the access dimensions. The new latent variables were baptized according to the items that loaded onto them the most. For example, 6 new/latent variables were generated for the physical access dimension. The items that loaded onto the second latent variable in the physical access dimension are items C14 and C16. To get the name of this new variable, we merged the meaning of C14 and C16 and called the variable ''Social Connections''. This was done for all the new/latent variables across the four (4) access dimensions. These findings are summarized in figure 2.

For peer review only - http://bmjopen.bmj.com/site/about/guidelines.xhtml

Independent predictors of cross border health access

Separate regressions were run against the mean score of the outcome variable "overall ease of access of cross border health care" for all the access dimensions. In all regressions, the independent variables were the latent variables extracted using factors analysis. The regression analysis was also stratified for each of the 3 disease conditions.

For physical access, the model explained 36% variation, which varied between 38-40% depending on the stratification by the three disease conditions. The strongest independent predictor of ''overall ease of physical crossing for care'' was ''ease of border crossing''. For financial access/affordability, the model explained 31% variation, which varied between 21-44% depending on the disease condition. The strongest independent predictor of overall ''financial access/affordability was the inverse of ''self-funding'' (i.e. services being free). The regression details are summarized in table 3.

Qualitative findings:

From the qualitative analysis, two overarching themes emerged and these were reasons for crossing the border to seek health care and experiences on how border resident communities navigate barriers during cross border health care access.

Reasons for crossing the border to seek health care

The main push factors for crossing to seek care across the border were: 1) service delivery points were more accessible, 2) health services were more affordable, 3) availability of services needed, 4) acceptability of the services and 5) perceived higher quality of services**.**

Physical accessibility of the health services

The border residents preferred to access services that are closer to them. In instances where the nearest health facilities was on the opposite side of the border, the residents preferred to cross the border to access services rather than travelling long distances to access services in their own country;

> *"The reason I went to Uganda, it is because the health facilities where we get our vaccination (RUBAYA, CYUMBA and KANIGA) are more distant, and the transport to reach there is expensive"* - **Border resident, Gatuna-Rwanda.**

Having relatives and friends on the opposite side of the border was mentioned as a reason why most border residents preferred to seek care across the border since they had an opportunity of being taken care of by their relatives:

For peer review only - http://bmjopen.bmj.com/site/about/guidelines.xhtml

> *"My relative live there, so I went there because they could take care of me after giving birth …. I just crossed because I knew my family would receive me"*-**Border resident, Gatuna-Rwanda.**

Affordability of the health services

Health services being more affordable prompted study communities to seek healthcare by crossing the border. This was more common where national health insurance was established while "free services" policy was on the opposite side of the border or wanted to access services that are not covered by insurance at home countries. They further noted that accessing these services without insurance in their own country was more costly. One respondent noted:

> *"It all started when I was coming from the hospital … I needed medication to recover, but, the medicines I required, were not covered under my health insurance. So a friend of mine recommend me to go to Uganda side"*- **Border resident, Gatuna-Rwanda**.

Availability of the health services

The inadequacies of services in one's home country often prompted border resident communities to cross. Border residents who visited the health facilities in their home countries and did not get the services they required tended to cross the border to access these services;

> *"I took my grandchild because after visiting the hospitals here for three consecutive times and missing the vaccine, I was forced to travel to Uganda for the vaccine"*- **Border Resident, Busia-Kenya.**

Formal and informal referral to quality, nearer and cheaper health facilities prompted border residents to seek healthcare from health facilities in the opposite side of the border. Formal referrals, were arranged by a health worker while informal referrals were prompted by self, friends, relatives and other community members.

> *"I started feeling labor pains where I visited the district hospital but got students who were attending to patients. I was bleeding and was advised to go to MASAFU (Uganda side) for delivery"*- **Border resident, Busia-Kenya.**

Acceptability

The fear of being stigmatised by fellow community members was also found among a few respondents. This prompted HIV positive border residents to seek health care from health facilities across the border fearing that home communities would come to know about their HIV sero-status;

> *"When I discovered I was infected with HIV I was stigmatized and couldn't stand going to pick drugs as I could meet very many people I know. I was forced to go to Uganda and gained courage when picking the drugs"*-**Border resident, Busia-Kenya.**

For peer review only - http://bmjopen.bmj.com/site/about/guidelines.xhtml

Perceived quality of the services

Border residents who perceived that services from the opposite side of the border were of better quality than at home tried to seek healthcare from health facilities across the border;

> *"When I went to the Kenyan hospital they worked on me well and I came back safely. The good thing in Kenya is that any nurse can help you, she tells you where to start from, and she directs where to get the book and notes all things concerning the baby."*- **Border resident, Busia-Uganda**

Experiences on how border resident communities navigated barriers during cross border health care access

From the narration of experiences with household adults that had successfully sought care across the border, the key facilitators that enabled navigation of barriers emerged across the physical and affordability dimensions. Under physical dimension, the main facilitators were presence of informal routes, having official travel documents and knowing a similar language. While, under affordability the main facilitator was the ability to meet the cost of care across the border.

Presence of informal routes

The presence of informal routes locally known as *"panya roads"* facilitated access to health care services from the opposite side of the border. Many border resident communities were not allowed to cross at the formal crossing routes for health services and resorted to using informal routes:

> *"When I pass via the customs offices, they sometimes ask for money or arrest me but I was arrested once. Now, I normally pass via a panya (short cut route) (to Kenya side) to pick my medicine"* - **Border resident, Busia-Uganda.**

> *"Border officers send you back because all those services are available here....(in Rwanda). Actually, most people who need those services use shortcuts to cross. Furthermore, Ugandans who take HIV treatment here (in Rwanda) also pass through there (the informal route)"* – **Border resident, Gatuna- Rwanda**

Having official travel documents

Border residents who had their identification documents, such as; identity cards and passports were easily allowed to cross the border. Most of the border residents without travel documents were not allowed to cross the border and, in most instance, they had to make informal payments or bribes so as to be allowed to cross. Some respondents however mentioned that in some instances, travel documents were not a requirement for crossing the border and this facilitated health care access. One respondent remarked:

For peer review only - http://bmjopen.bmj.com/site/about/guidelines.xhtml

> *"No, whenever I visit there I am never asked for any document either when going or coming back*"- **Border Resident, Busia-Kenya.**

Similarity in language

Knowing the local dialect of the neighbouring border communities facilitated access to health care services across the border. Border residents were able to easily communicate with the service providers and even to disguise as nationals of the neighbouring country. On the other hand, the inability to speak the local language hindered access to health care services due to language barrier and sometimes had to get translators.

> "*I was just treated well after paying the money required. It's only the language barrier that made the whole process look tiresome*"**-Border Resident, Isebania-Kenya**.

Ability to meet the cost of care

The cost of care included the money paid for the actual health services, the transport costs and other informal payments or bribes made to facilitate the process of accessing care. The respondents who had crossed to access care on the opposite side of the border unanimously agreed that their ability to pay the actual cost of services enables them to access health services from there. In most cases, HIV treatment and childhood immunisation services were free and this enabled border residents with these conditions to access care.

> *"The cost was so much reasonable as compared to Kenyans side and we also used Kenyan currency which is stronger that the Uganda shilling"*- **Border Resident, Busia-Kenya.**

Relatedly, being able to pay informal payments/ bribes either at the border point (to border officials) or at the health facility enabled access to health services at the opposite border

> *"While on the way we encountered the police whom we bribed to proceed with our journey to Dabani Hospital. At the hospital we had a midwife who was attending to her by bringing her food and water to bath and we ended up paying her (the midwife)"***- Border Resident, Busia-Kenya.**

Discussion

This study aimed to explore how to increase health care access for communities along the border in East Africa. We found that communities resident near national borders were able to cross borders to seek health care. There are factors that attract border residents to seek care across the border and these centred on service delivery points being near, availability of services and health services being of acceptable quality across the border compared to the home country. From the quantitative analysis the main facilitators for cross border health access

For peer review only - http://bmjopen.bmj.com/site/about/guidelines.xhtml

were ease of physically crossing the border and health services being affordable across the border among other factors.

Rather than travelling long distances to access healthcare in the home country, border residents preferred to access services that were closer to them though across the border. This has also been seen elsewhere [24] that patients gravitate to their nearest convenient health service provider. Other than physical "closeness" of the health care across the border, many border residents in our study commonly used informal routes "*Panya roads*" to cross for health care – an indication of porous borders referred to as "ease of border crossing". The ease of physical access is also enhanced by the fact that for many of the border communities, the culture and language is the same and they live as one community irrespective of the border [15]. This is evidenced from the respondents' remarks that knowledge of the local language and family relatives that reside across the border facilitated crossing for health care.

Border residents chose to seek health care across the border because it was affordable compared to care within their home country. Studies done among U.S. residents living along the U.S.–Mexico border have shown that due to the economic, financial, and health care access barriers, border residents on the US side commonly resort to crossing into Mexico to meet their health care needs because of much more affordable prescription medications as well as services [6,7]. For the border residents in East Africa, there seemed to be an opportunity cost they were willing to pay in terms of other costs like transport and informal payments at border crossing. However, we acknowledge that this opportunity cost was easier for the border residents to bear since they were seeking services like childhood immunization and HIV treatment refill which are usually free of charge in East Africa [25]. Apart from affordability in terms of cost of care, border residents who lacked health insurance also preferred to seek care across the border where national health insurance was not being implemented. There is need for East African countries to have common arrangements (insurance or free care) that especially help poor communities along the border to access care. Although the non-insured community members can access care across the border, this leaves them disadvantaged in case the service needed is only available in their home country [26].

Some border residents also preferred to go where care was of acceptable quality. This was more important for mothers seeking delivery services and HIV patients that preferred confidentiality.

For peer review only - http://bmjopen.bmj.com/site/about/guidelines.xhtml

This speaks to the need for countries to improves service quality so that citizens in remote areas are more willing to use the available services at home [27].

From our study, we see the need to move from nationally circumscribed rights and entitlements to a legal environment that allows border residents to freely access care cross the border if the nearest service delivery point is on the opposite side of the border. Though border residents are individually navigating access barriers, this leaves them vulnerable if some legal frameworks were to be enforced. For UHC to be efficiently delivered, countries may need to revise their planning, resourcing and regulatory arrangements to enable border communities access the existing healthcare provision without bearing the hardship of navigating these barriers on their own [20]. This can also reduce the uncertainty and discretion of front-line officials if they were all aware of the access rights for border residents in neighboring communities. This can span to other cross border health issues such as having joint efforts to deal with disease outbreaks[28,29].

At regional level, centers of excellence are beginning to form with a view of serving people from the EAC [30] and there is need for innovative arrangement at micro level for efficient delivery of health care. It would be useful to share the available healthcare resources (for example, every side of the border does not have to build a hospital if one side of the border has one in close distance). Guidelines should be developed to enable the use of a common pool of health care resources and optimize efficiency on both sides of the border. The European region has tried to remove legal and administrative barriers through cross-border cooperation (CBC) [31] aimed at "identifying potential complementarities in all fields of human activities in the border regions to ease the lives of the border residents". There are lessons that can be driven from these and other cross-border integration initiatives [13].

As this paper goes to publication, cross border health has been made more urgent by the Corona virus pandemic and its impact on the border health systems to provide testing and surveillance for interstate truck drivers in East Africa. The guidelines to manage the pandemic have come into sharp confrontation with the objectives for legal rights of interstate travel and movement of goods – in some cases threatening to dismantle the regional cooperation mission [32]. Numerous media carried stories showing long queues of trucks some extending 20 Km at the border all waiting for Corona virus testing that was sometimes taking 2-3 days to offer the test results and clear the truck through emigration points. Communities around these long jams of

For peer review only - http://bmjopen.bmj.com/site/about/guidelines.xhtml

trucks were also exposed to community transmission of coronavirus from the truck drivers – a group that had a high occupational risk of contracting and transmitting the virus [33]. This further demonstrates the need to invest in and coordinate better the economic and health care objectives at border points for the East African community to consolidate its integration and social economic functionality.

Study limitations:

This paper only presents the views of respondents that successfully navigated access barriers. Our study sites were the official cross points. We did not include the numerous informal cross points which may have different contexts and experiences. The use of official crossing was selected to enable the study to factor in the operations of the formal rules for emigration when seeking health care across the border.

Conclusion

From this study, we note that communities resident near national borders of the EAC were able to cross borders to seek health care. Although the EAC integration agenda has been discussed for a while, progress has been made in the areas of economics and trade and it has not yet benefited border residents in terms of access to healthcare across the border. There is need for: innovative arrangements to optimize the existing resources and a policy environment to enable East Africa invest better and realize synergies for the Communities resident at the borders for health integration in the EAC.

Acknowledgments

The authors thank the District or County Leadership at the following border sites: Katuna-Uganda (Kabale District); Gatuna-Rwanda (Gicumbi District); Rusomo-Rwanda (Kibehe District); Busia-Uganda (Busia District); Busia-Kenya (Busia County); Isebania-Kenya (Migori County). Thanks to Makerere University School of Public Health, SPEED Project and many thanks to the following persons for their key role in the data collection activities: Mr Moses Sitati, Researcher at Moi University who led the field team in Kenya, Shadat Kititi of Makerere University School of Public Health who led the field team in Uganda and Vincent Mutabazi of University of Rwanda who led the field team in Rwanda.

Contributors

For peer review only - http://bmjopen.bmj.com/site/about/guidelines.xhtml

FS, DT, SS, SB, AS and MN conceptualised the study. FS, DT and SB led the team's work. FS, LM and MN sought country level study approvals. FS, DT, SB, SS, AS, MA, AR, LM and MN led the study pre-visits and date collection. FS, SS, MA and AS led the quantitative data analysis while DT, SB, AR, LM and MN led the qualitative data analysis. FS, DT, SS and MA wrote the draft manuscript, with all authors providing feedback and approving the final version

Ethics approval and consent to participate: Ethical approval for this study was provided by Makerere University Higher Degree Research and Ethics Committee (HDREC Protocol number 583) and the Uganda National Council of Science and Technology (Reg No SS4713). In Kenya the study was approved by Moi University/Moi Teaching Referral Hospital Research and Ethics Committee (FAN: IREC: 3077); in Rwanda was approved by the Rwanda National Ethics Committee (No.438/RNEC/2018) and in Tanzania by the University of Dar es Salaam Research Ethics Committee (Reference Number UDSM-REC/2018/02). All study respondents provided informed consent prior to participating in the study.

Consent for publication: Not applicable.

Availability of data and materials: The datasets used and/or analyzed during for our study are available from the corresponding author upon reasonable request.

Competing interests: The authors declare that they have no competing interests

Funding: This work was supported by MRC/Wellcome Trust, UK, grant number: MR/R02028/1

For peer review only - http://bmjopen.bmj.com/site/about/guidelines.xhtml

References

1. Mansourian BP. *Global Perspectives in Health-Volume II.* Vol 3: EOLSS Publications; 2009.

2. Rodríguez-Saldaña J. Challenges and opportunities in border health. *Preventing chronic disease.* 2005;2(1).

3. United States Mexico Border Health Commission. Border lives: health status in the United States-Mexico border region. *El Paso (TX): United States–Mexico Border Commission.* 2010.

4. Legido-Quigley H, Glinos I, Baeten R, McKee M. Patient mobility in the European Union. *Bmj.* 2007;334(7586):188-190.

5. Ried W, Marschall P. Potential benefits of crossborder health care: The case of Poland and Germany. *Economics & Sociology.* 2016;9(3):14.

6. Miller-Thayer J. Health migration: crossing borders for affordable health care. *Field Actions Science Reports. The journal of field actions.* 2010(Special Issue 2).

7. Su D, Richardson C, Wen M, Pagán JA. Cross-border utilization of health care: evidence from a population-based study in south Texas. *Health services research.* 2011;46(3):859-876.

8. Levesque J-F, Harris MF, Russell G. Patient-centred access to health care: conceptualising access at the interface of health systems and populations. *International journal for equity in health.* 2013;12(1):18.

9. Hanson K, Ranson MK, Oliveira-Cruz V, Mills A. Expanding access to priority health interventions: a framework for understanding the constraints to scaling-up. *Journal of International Development: The Journal of the Development Studies Association.* 2003;15(1):1-14.

10. Andersen R, JF N. Andersen and Newman framework of health services utilization. *Journal of Health and Social Behavior.* 1995;36:1-10.

11. Bruhn JG. *Culture and health disparities: evaluation of interventions and outcomes in the US-Mexico border region.* Springer; 2014.

12. Hunter-Adams J, Rother H-A. A Qualitative study of language barriers between South African health care providers and cross-border migrants. *BMC Health Services Research.* 2017/01/31 2017;17(1):97.

13. Böhm H, Kurowska-Pysz J. Can Cross-Border Healthcare Be Sustainable? An Example from the Czech-Austrian Borderland. *Sustainability.* 2019;11(24):6980.

14. Van Reisen M, Mawere M, Stokmans M, Gebre-Egziabher KA. *Roaming Africa: Migration, Resilience and Social Protection.* Vol 2: Langaa RPCIG; 2019.

For peer review only - http://bmjopen.bmj.com/site/about/guidelines.xhtml

15. Durham J, Blondell SJ. A realist synthesis of cross-border patient movement from low and middle income countries to similar or higher income countries. *Global Health.* 2017;13(1):68-68.

16. Leontidou L, Afouxenidis A, Donnan H, et al. Border Cities and Towns: Causes of Social Exclusion in Peripheral Europe. Brussels: European Commission. Targeted Socio-Economic Research (TSER) SOE2 …; 2001.

17. Maessen JH, Raab J, Haverkate M, et al. How prepared are we for cross-border outbreaks? An exploratory analysis of cross-border response networks for outbreaks of multidrug resistant microorganisms in the Netherlands and Germany. *PloS one.* 2019;14(7).

18. Nsoedo EE. The Ebola Crisis in the West African region: should it have been so severe? *Open Journal of Social Sciences.* 2014;2(10):98.

19. Wetzel D. Pandemics know no borders: In Africa, regional collaboration is key to fighting COVID-19

2020; https://blogs.worldbank.org/africacan/pandemics-know-no-borders-africa-regional-collaboration-key-fighting-covid-19. Accessed 24th June 2020.

20. UN. *Transforming Our World: The 2030 Agenda for Sustainable Development.* United Nations;2015.

21. deMarrais KB, Lapan SD. *Foundations for research: Methods of inquiry in education and the social sciences.* Routledge; 2003.

22. Hsieh H-F, Shannon SE. Three approaches to qualitative content analysis. *Qualitative health research.* 2005;15(9):1277-1288.

23. Costello AB, Osborne J. Best practices in exploratory factor analysis: Four recommendations for getting the most from your analysis. *Practical assessment, research, and evaluation.* 2005;10(1):7.

24. Welsh Affairs Committee. The Provision of Cross-border Health Services for Wales. *London, The Stationery Office,(HC 56 of 2008–09).* 2008.

25. MEASURE Evaluation. *East Africa Cross Border Integrated Health Study.* The Carolina Population Center, University of North Carolina 2017. 978-1-9433-6469-5.

26. Busse R. Border-crossing patients in the EU. *EUROHEALTH-LONDON-.* 2002;8(4):19-21.

27. Suñol R, Garel P, Jacquerye A. Cross-border care and healthcare quality improvement in Europe: the MARQuIS research project. *Qual Saf Health Care.* 2009;18 Suppl 1(Suppl_1):i3-i7.

28. WHOAFRO. Ten African countries endorse cross-border collaboration framework on Ebola outbreak preparedness and response. 2019; https://www.afro.who.int/news/ten-african-countries-endorse-cross-border-collaboration-framework-ebola-outbreak-preparedness. Accessed 16th May, 2020.

For peer review only - http://bmjopen.bmj.com/site/about/guidelines.xhtml

29. Olu OO, Lako R, Wamala JF, et al. What did we learn from preparing for cross-border transmission of Ebola virus disease into a complex humanitarian setting – The Republic of South Sudan? *Infectious Diseases of Poverty.* 2020/04/21 2020;9(1):40.

30. EASTECO. *Concept Note: THE SECOND EAC REGIONAL E-HEALTH & TELEMEDICINE WORKSHOP, MINISTERIAL CONFERENCE AND TRADE EXHIBITION, Kigali, Rwanda, 15-18 May 2018.* The East African Science and Technology Commission;2018.

31. EC. *Easing legal and administrative obstacles in EU border regions.* Luxembourg: European Commission;2017.

32. Victor B. Stop Criminalising Truck Drivers In War Against COVID-19. 2020; 13th June 2020. Available at: https://www.capitalfm.co.ke/news/2020/05/stop-criminalising-truck-drivers-in-war-against-covid-19/, 2020.

33. Address by H.E. Yoweri Kaguta Museveni, President of the Republic of Uganda to the nation: Latest Updates on Matters Regarding Corona Virus (COVID 19) [press release]. Kampala: The State House of Uganda, 4th May 2020 2020.

For peer review only

Table 1: Socio-demographic characteristics of the household adults who reported on household members who had ever crossed the border to seek healthcare services

Characteristic	Frequency (n)	Percentages (%)
Sex		
Male	28	10.0
Female	251	90.0
Age (Mean, SD)	Mean = 32.8	SD = 9.8
Educational Level		
No Education	35	12.5
Some Primary	107	38.4
Completed Primary	48	17.2
Some Secondary	49	17.6
Completed Secondary	27	9.7
Tertiary	13	4.7
Marital Status		
Single	28	10.0
Married	213	76.3
Divorced	16	9.3
Widowed	12	4.3
Employment Status		
Not Employed	131	47.3
Informal & Self Employment	135	48.7
Formal employment	11	4.0
Nationality		
Kenyan	82	31.1
Rwandese	89	33.7
Tanzanian	6	2.3
Ugandan	86	32.6
Dual Citizenship	1	0.4
Has relatives on opposite border		
Yes	207	75.0
No	69	25.0
Border site		
Busia Kenya-Busia Uganda	97	34.8
Gatuna-Katuna	91	29.0
Isebania	49	17.6
Rusumu	52	18.6
Country of residence		
Kenya	84	30.1
Rwanda	101	36.2
Uganda	94	33.7

For peer review only - http://bmjopen.bmj.com/site/about/guidelines.xhtml

Table 2: Characteristics of border residents who accessed health care from the opposite side of the border

Characteristic	Frequency (n)	Percentages (%)
Age (Mean, SD)	Mean = 22.7	SD = 16.7
Sex		
Male	74	26.5
Female	205	73.5
When care was sought		
< 1 Month	22	22.9
1 to 3 Months	58	20.8
>3 to 6 Months	26	9.3
>6 to 12 Months	54	19.4
>12 Months to 5 years	77	27.6
Type of facility where care was sought		
Public	234	83.9
Private Not For Profit	11	3.9
Private For Profit	32	11.5
Don't Remember	2	0.7
Service received		
Delivery	98	35.1
HIV treatment	77	27.6
Immunisation	104	37.3
Type of service received		
Diagnosis Only	3	1.1
Treatment as OPD	192	68.8
Admission	40	14.3
Operation	44	15.8
Reasons why care was sought		
Better quality	99	36.0
Referral	10	3.6
Less Costly	79	28.7
Community Support	18	6.6
Stigma	12	4.3
Proximity	25	9.0
Other	51	18.6

For peer review only - http://bmjopen.bmj.com/site/about/guidelines.xhtml

Table 3: Independent predictors of overall ease of cross border access to health-care

Access dimension	**Regression model items**	**Regression coefficients**			
		Overall	Delivery	HIV	Immunization
Physical Access	Constant	3.221	3.23	3.15	3.254
	Ease of border crossing	**0.381**	**0.346**	**0.565**	**0.371**
	Social connections	-0.79	-0.09	-0.035	-0.05
	Satisfaction with organization and approachability of facilities at home	-0.006	-0.012	0.126	-0.105
	Documentation of referral for health care needs	**0.098**	0.146	0.034	0.133
	Satisfaction with the organization and approachability of facilities across the border	**0.156**	**0.164**	0.123	**0.217**
	Distance to facility	**0.224**	**0.213**	**0.316**	**0.226**
	R square	0.361	0.379	0.402	0.388
	N	251	89	69	91
Financial access/ Affordability	Constant	3.009	2.918	2.78	3.027
	Self-funded	**-0.478**	**-0.496**	**-0.646**	**-0.387**
	Time & transport cost acceptable	0.004	-0.075	**0.488**	-0.049
	Cost of care affordable	**0.324**	**0.522**	0.147	**0.260**
	Insurance needed	0.006	0.018	**-0.233**	0.154
	Local currency and financial support	-0.047	-1.13	0.066	-0.049
	R square	0.305	0.437	0.437	0.213
	N	248	95	62	89
Availability/ Accommodation	Constant	3.451	3.443	3.487	3.411
	Better workforce	**0.106**	0.045	**0.216**	0.108
	Provider Public-Private mix	0.064	0.047	0.002	0.103
	Services/medicines available	**0.274**	**0.297**	**0.393**	**0.144**
	Not getting better at home	0.04	0.023	0.063	0.033
	Language was not a barrier	0.021	0.011	-0.01	0.061
	R square	0.242	0.295	0.471	0.113
	N	252	90	70	90
Acceptability and Appropriateness	Constant	3.395	3.37	3.311	3.462
	Acceptable Quality	**0.364**	**0.388**	**0.372**	**0.325**
	Accountability	**0.142**	**0.154**	**0.228**	0.062
	R square	0.364	0.462	0.396	0.246
	N	262	92	73	95

For peer review only - http://bmjopen.bmj.com/site/about/guidelines.xhtml

Figure 1: Study sites (indicated by red circles)

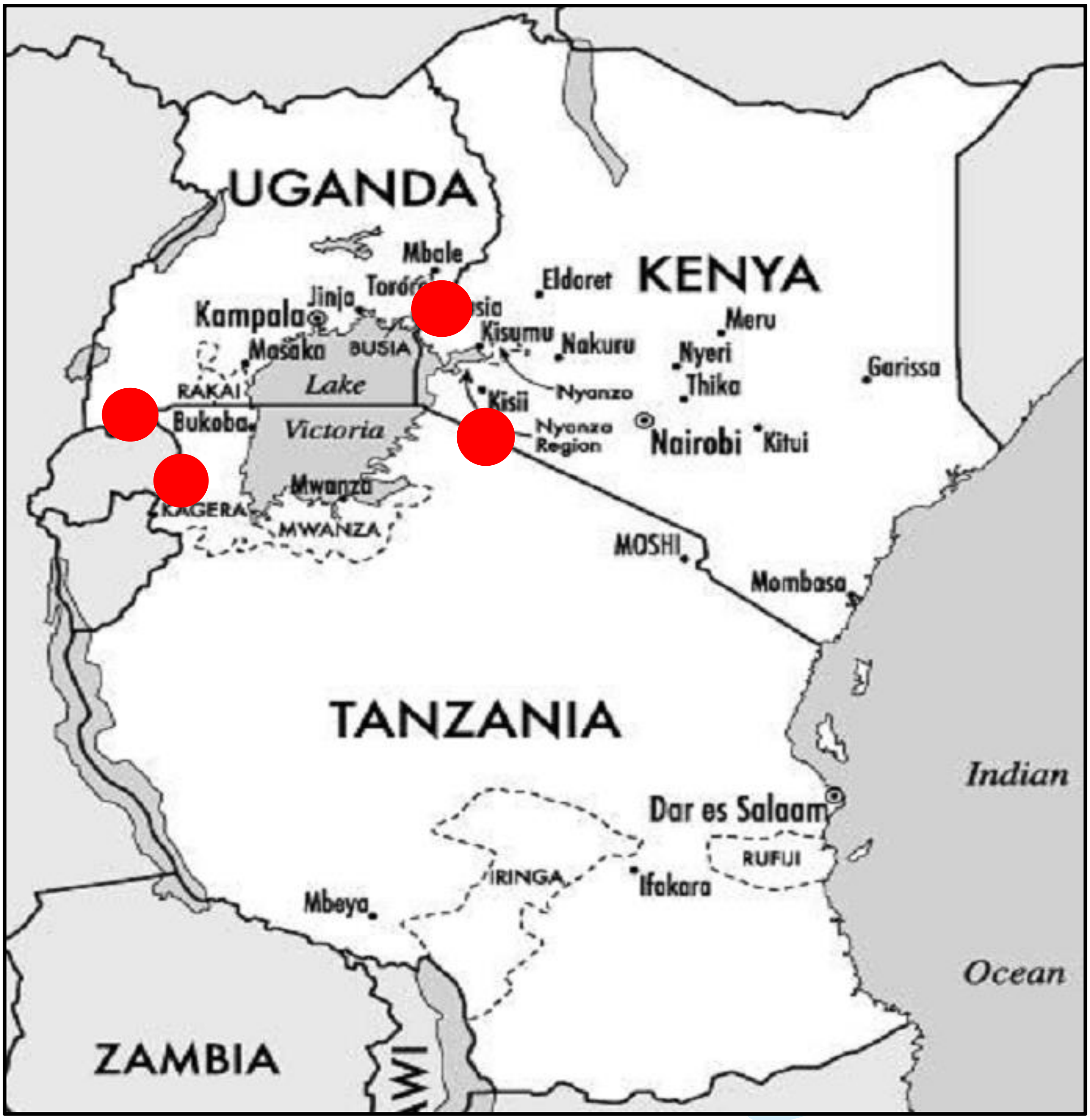

Figure 2: New generated variables for each of the 4 studied access dimensions and corresponding items that loaded with a factor of 0.7 and above onto the new variables

Physical access Dimension

Items	Latent variables/Item loadings					
	Ease of crossing the border	Social Connections	Satisfaction with organization of service at home	Referral Documents	Satisfaction with organization of services across the border	Distance to the health facility
c1						.860
c2	.702					
c3	.756					
c5			.873			
c6			.905			
c7					.857	
c8					.822	
c10	.728					
c12				.751		
c13				.739		
c14		.770				
c16		.707				

Financial access/ Affordability Dimension

Ite ms	Latent variables/Item loadings				
	Self-funded	Time & transport cost acceptable	Cost of care affordable	Insurance needed	Local currency and financial support
d1			.745		
d3		.875			
d4		.832			
d5	.862				
d7	-.824				
d9					.793
d10	.825				
d11				.826	

Availability and Accommodation Dimension

	Latent variables/Item loadings				
	Better Workforce	Provider Public-private mix Across	Services & medicines available Across	Not getting better at home	Language was not a barrier
e1				0.797	
e2				0.717	
e4			0.842		
e5	0.855				
e6	0.825				
e8			0.791		
e10					
e11		0.709			
e13		0.718			
e16					0.835

Acceptability and Appropriateness Dimension

	Latent variables/Item loadings	
Items	Acceptable Quality	Accountability
f1	.900	
f2	.911	
f3		
f4		.858
f5		.811

Figure 3: The extent to which each access dimension contributed to the ease of accessing healthcare from the opposite side of the border for each of the 3 disease conditions

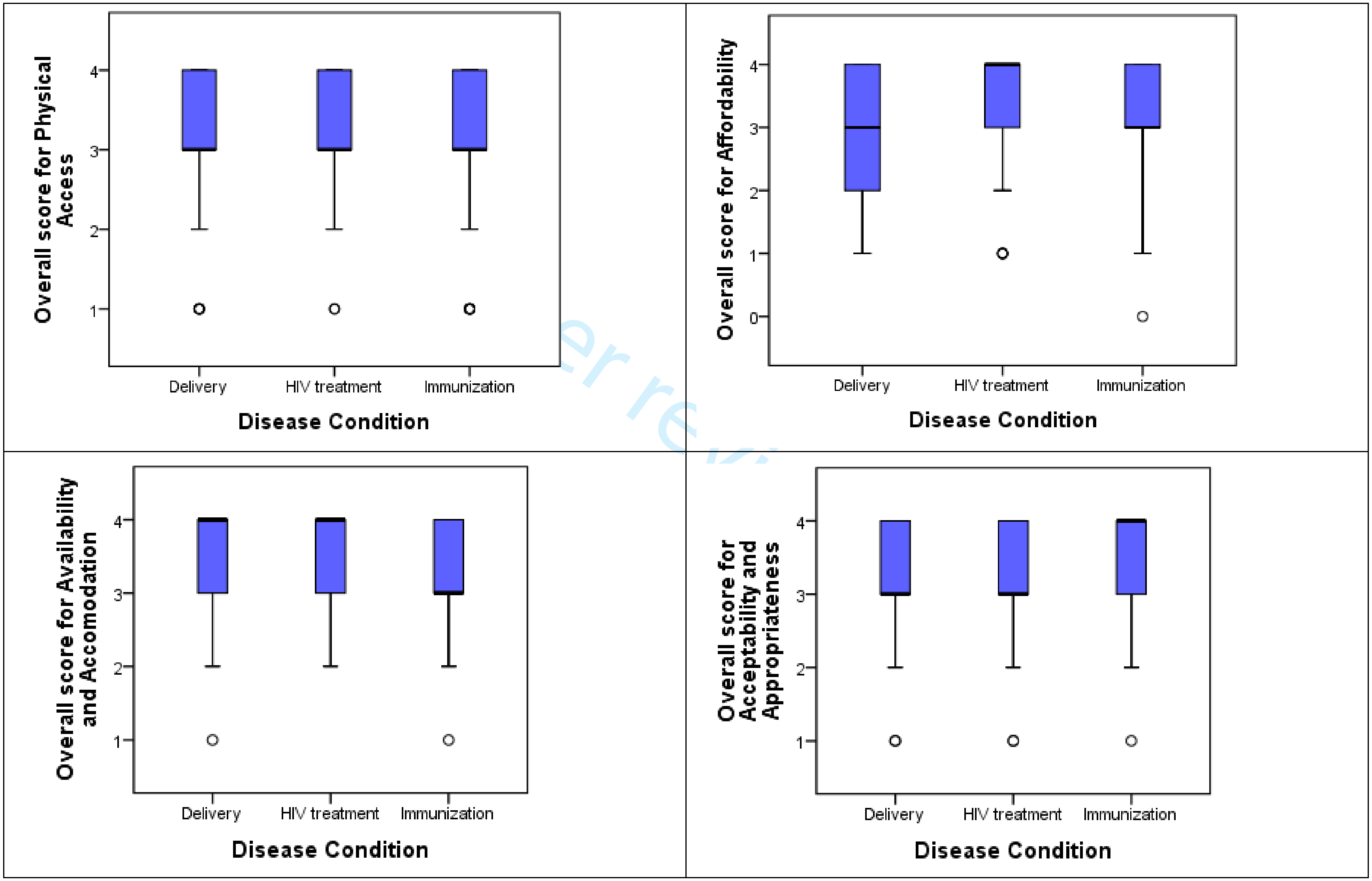

BMJ Open

Experiences of seeking health care across the border: Lessons to inform upstream policies and system developments on cross border health in East Africa

Journal:	*BMJ Open*
Manuscript ID	bmjopen-2020-045575.R1
Article Type:	Original research
Date Submitted by the Author:	28-Sep-2021
Complete List of Authors:	Ssengooba, Freddie; Makerere University, School of Public Health, Department of Health Policy Planning and Management; Makerere University School of Public Health, SPEED Project<br>Tuhebwe, Doreen; Makerere University School of Public Health, Department of Health Policy Planning and Management<br>Ssendagire, Steven; Makerere University School of Public Health, Department of Health Policy Planning and Management<br>Babirye , Susan ; Makerere University School of Public Health, Department of Health Policy Planning and Management<br>Akulume , Martha ; Makerere University School of Public Health, Department of Health Policy Planning and Management<br>Ssennyonjo, Aloysius; Makerere University School of Public Health, Department of Health Policy Planning and Management; Makerere University School of Public Health, SPEED Project<br>Rutaroh, Arthur; African Health Economics and Policy Association.<br>Mutesa, Leon; University of Rwanda, College of Medicine and Health Sciences<br>Nanagami, Mabel; Moi University College of Health Sciences, Health Policy and Management
**Primary Subject Heading**:	Health services research
Secondary Subject Heading:	Public health
Keywords:	Public health < INFECTIOUS DISEASES, PUBLIC HEALTH, Quality in health care < HEALTH SERVICES ADMINISTRATION & MANAGEMENT

SCHOLARONE™
Manuscripts

I, the Submitting Author has the right to grant and does grant on behalf of all authors of the Work (as defined in the below author licence), an exclusive licence and/or a non-exclusive licence for contributions from authors who are: i) UK Crown employees; ii) where BMJ has agreed a CC-BY licence shall apply, and/or iii) in accordance with the terms applicable for US Federal Government officers or employees acting as part of their official duties; on a worldwide, perpetual, irrevocable, royalty-free basis to BMJ Publishing Group Ltd ("BMJ") its licensees and where the relevant Journal is co-owned by BMJ to the co-owners of the Journal, to publish the Work in this journal and any other BMJ products and to exploit all rights, as set out in our licence.

The Submitting Author accepts and understands that any supply made under these terms is made by BMJ to the Submitting Author unless you are acting as an employee on behalf of your employer or a postgraduate student of an affiliated institution which is paying any applicable article publishing charge ("APC") for Open Access articles. Where the Submitting Author wishes to make the Work available on an Open Access basis (and intends to pay the relevant APC), the terms of reuse of such Open Access shall be governed by a Creative Commons licence – details of these licences and which Creative Commons licence will apply to this Work are set out in our licence referred to above.

Other than as permitted in any relevant BMJ Author's Self Archiving Policies, I confirm this Work has not been accepted for publication elsewhere, is not being considered for publication elsewhere and does not duplicate material already published. I confirm all authors consent to publication of this Work and authorise the granting of this licence.

For peer review only

Experiences of seeking health care across the border: Lessons to inform upstream policies and system developments on cross border health in East Africa

Authors: Freddie Ssengooba*[1,2], Doreen Tuhebwe[1], Steven Ssendagire[1], Susan Babirye[1], Martha Akulume[1], Aloysius Ssennyonjo[1,2], Arthur Rutaroh[3], Leon Mutesa[4], and Mabel Nangami[5]

[1]. Department of Health Policy Planning and Management, Makerere University School of Public Health, Kampala, Uganda
[2]. Makerere University School of Public Health, SPEED Project
[3]. African Health Economics and Policy Association, Kampala, Uganda
[4]. University of Rwanda, College of Medicine and Health Sciences Kigali, Rwanda
[5]. Department of Health Policy and Management, Moi University College of health Sciences, Eldoret, Kenya

*Corresponding author, sengooba@musph.ac.ug or freddie.ssengooba@gmail.com +256772509316

Key words: cross border, border residents, health access, universal health coverage, East Africa

Word count: 6286 (abstract, strengths, limitations, introduction, methods, results, discussion and conclusion)

Abstract

Objectives: This study explored the experiences of accessing care across the border in East Africa.

Participants: From February-June 2018, a cross-sectional study using qualitative and quantitative methods was conducted among 279 household adults residing along selected national border sites of Uganda, Kenya and Rwanda and had accessed care from the opposite side of the border 5 years prior to this study.

Setting: Access to HIV treatment, maternal delivery and childhood immunization services was explored. We applied the health access framework and an appreciative inquiry approach to identify factors that enabled access to the services.

For peer review only - http://bmjopen.bmj.com/site/about/guidelines.xhtml

Measures: Exploratory Factor Analysis and linear regression were used for quantitative data, while deductive content analysis was done for the qualitative data on respondent's experiences navigating health access barriers.

Results: The majority of respondents (88.9%; 234/279) had accessed care from public health facilities. Nearly one third (77/279) had sought care across the border more than a year ago and 22.9% (64/279) less than a month ago. From the linear regression, the main predictor for ease of access for health care were ''ease of border crossing" (Regression Coefficient (RegCoef) 0.381); "services being free" (RegCoef 0.478); "services and medicines availability" (RegCoef 0.274) and "acceptable quality of services" (RegCoef 0.364). The key facilitators for successful navigation of access barriers were related to the presence of informal routes, speaking a similar language and the ability to pay for the services.

Conclusion: Communities resident near national borders were able to cross borders to seek health care. There is need for a policy environment to enable East Africa invest better and realize synergies for these communities. This will advance Universal Health Coverage goals for communities along the border who represent the far fang areas of the health system with multiple barriers to health care access.

Strengths and limitations of this study

- The study involved the use of both qualitative and quantitative data which strengthened the triangulation of results.
- Having multi country study sites in the East Africa Community strengthened the study.
- This paper only presents the views of respondents that successfully navigated access barriers.
- We did not include communities along the numerous informal cross points which may have different contexts and experiences.

For peer review only - http://bmjopen.bmj.com/site/about/guidelines.xhtml

Introduction

Border resident communities are the population settlements, rural or urban, in close proximity to the borders[1]. This term usually applies to international borders and the border resident communities of the two neighbouring countries are usually economically and socially interdependent [2] and have more in common with their sister communities across the border than with communities on their own side of the border. Residents of both countries cross the border for a variety of reasons, including; work, shopping, health care, visits with friends or relatives, and others [3].

Increasingly, border resident communities cross to access care in each other's country [4]. This is in partly due to challenges of accessing the needed care in the home country where the nearest health facility may be far away into their country and the border residents are located at the periphery with physical and economic access barriers. Cross-border health care can also enhance individual choice, by providing added option to obtain health care which may either exhibit a better quality or be available at a lower cost in comparison with their national provision system [5,6]. A study by Su and colleagues showed that one of the most significant predictors of health care utilization in Mexico by US citizens were lack of US health insurance coverage and dissatisfaction with the quality of care in the US[7]. Scholars have also proposed several frameworks that can be used to understand facilitators and barriers to health access[8-10].

However, access to care in the neighbouring country may not always be easy given the several barriers experienced along the pathway to care[2]. Some of the barriers to physical access may include challenges of poor road network and terrain and lack of identity documentation[11]. It could be an issue of financial barriers like high cost of care across the border despite of physical proximity, need for foreign currency and un-transferability of health insurance policy to a neighbouring country[5]. Other hindrances include; lack of quality health care infrastructure, health care workforce shortages and language barriers that pose acceptability challenges [3,12]. These barriers to access and utilization are often times rooted in the differences within legal and institutional frameworks that confer entitlement to health care between countries as well as creating differences in health care protocols [13].

Although states have obligations to ensure their citizens have access to all health services [14], this is sometimes not fulfilled especially for communities residing along international borders[15]. Because, the nearest health facility or health services needed may be across the border, individuals and communities usually navigate these barriers in order to access care in the neighbouring country [16] though not institutionally recognised or planned for by the country

For peer review only - http://bmjopen.bmj.com/site/about/guidelines.xhtml

receiving the clients. In the context of the East African Community (EAC), member states (Uganda, Kenya, Tanzania, Rwanda, Burundi and South Sudan) have a progressive plan to increase the interaction and freedom of movements of commercial goods and people.

Literature has shown several mechanisms that border residents use to navigate health access barriers. A few of the strategies include using social and kinship networks which facilitate cross-border exchange of information about where care is available and where quality can be accessed [6,11]. This literature also shows that people take advantage of the cultural context and the ethno-linguistic similarities to navigate barriers such as ineligibility or special preconditions for noncitizens to health care services[17]. Because some of the border residents live as one community in the two opposite countries, there are usually many informal crossings through non-official crossing points for trade and inherently promote cross border health access.

Border communities, regardless of their size, are often regarded as peripheral in terms of social programs but paradoxically have high priority in terms of national security and in allocation of citizenry privileges[18]. Of recent, border communities have also become of importance for public health surveillance due to threats like multi-drug resistant TB[19], Ebola outbreak experience in West Africa[20] and recently, the novel corona virus disease (COVID-19). These threats can and have traversed borders as a result of inflows and outflows of people and goods across borders. The disease surveillance objectives have in some places forced health systems across the borders to cooperate in the control of disease outbreaks[21]. It is not clear how this cooperation is extending to support day-to-day health care needs of communities along the borders. As another paper that we are working on demonstrates, there are major elements of state obligations in ensuring access to health for those in need since "health is a right". However, these obligations are limited to citizens apart from a few aspects like epidemic control events and the high end chronic care centers of excellence that are part of the regional efforts to address cross border health access.

Leaving no one behind is the mantra for Universal Health Coverage (UHC) and the Sustainable Development Goals (SDGs)[22]. Communities like those residing along the EAC borders must be targeted because they represent the far fang areas of the health system with multiple barriers to health care access. Beside distance from cities and towns, they also face administrative barriers arising from citizenship and movement to reach functional services – especially if from the other country. If not well articulated in policies and arrangements of health service provision, populations along the borders may remain vulnerable to poor access to health care

For peer review only - http://bmjopen.bmj.com/site/about/guidelines.xhtml

and at risk of being left behind. This study set out to document experiences and feasible strategies to advance access and UHC goals especially for communities residing along the EAC borders. This study addressed two questions; 1) why border residents cross to seek health care across the border and 2) how they navigate institutional health system barriers and challenges of seeking health care across the border (physical, financial, availability and acceptability) in East Africa.

Methods

Study setting and sites

This study was conducted in four land-cross border sites in East Africa in the countries of Uganda, Rwanda and Kenya. These sites are; 1) Katuna-Uganda/Gatuna-Rwanda, 2) Rusomo-Rwanda/Lusomo-Tanzania, 3) Busia-Uganda/ Busia-Kenya and 4) Isebania-Kenya/ Sirare-Tanzania. The physical location of the study sights is shown in figure 1 [caption: Study sites (indicated by red circles)]. These border sites are mostly remote and peasant with a few trading centers acting like border posts. Only Busia (Uganda-Kenya border) is a large urban trading site. At the formal border posts there are small business enterprises to promote cross border trade. The sites are usually located within a district (Uganda and Rwanda) or a county (Kenya). The districts or county are served by a local health facility that should provide a minimum health package to the citizens as mandated by the government. At these local health facilities, the services are only planned and budgeted to serve the citizens. In Rwanda all citizens have health insurance and one can only receive care under insurance. The study sites have some differences in the level of care that can be provided for example, while in Busia-Uganda there is a health center three functioning under a "free health care policy", in Busia-Kenya, there is a hospital (is higher than a health center three) and some services can only be accessed through pay by non-citizens.

Study design and respondent population

A cross-sectional design was used with quantitative and qualitative data collection methods. The study participants were the border resident communities at the selected border sites. We defined a border resident community as that community within 5-10 kilometers on either side of a designated international border crossing point, depending on whether the community is clustered at the exact crossing point or along the main highway that transits through the border crossing point. This study collected data around there case conditions that were selected to provide diverse experience in terms of 1) chronic care (HIV treatment), emergency care

For peer review only - http://bmjopen.bmj.com/site/about/guidelines.xhtml

(maternal delivery) and prevention (childhood immunization). These three cases also represent typical services that are part of the health care packages in all East African countries.

Sample size and sampling

At each study site, 75 survey respondents were targeted for survey and were divided equally across either side of the border for the three case conditions under study (i.e. a total of 300) as a purposeful sample that could ensure we collected as many diverse views as possible given the resources that we had. This study realized a sample of 279 household adults. To identify potential survey respondents, we asked the in-charges of the major catchment health facility on each of the study site to invite household adults in the health facility catchment area for a community dialogue. During the dialogue, the household adults (or any of their household members) who had ever accessed care from the opposite side of the border in the past five years were identified.

Upon identification, each of the household adult was secretly screened by the researcher to identify which care they or their household member(s) had accessed from the opposite side of the border. If the household adult or their household member(s) had ever accessed HIV treatment refill, maternal delivery and/or childhood immunization services from the opposite side of the border within 5 years prior to this study, they were consented for an interview about their experience accessing care across the border. The process of confidential screening was repeated until the targeted number of respondents for each case condition was realized.

Questionnaire Development and Data collection

From February-June 2018, an interviewer administered semi-structured questionnaire was used to survey the respondents. During the interview, questions were sequenced [23] to first explore the narration of the respondents' experience seeking care followed by the Likert-style survey questions about the perceived ease of access across the health access dimensions and finally the reasons for the stated score.

A questionnaire was developed specifically for this study. The design was informed by the health access framework [8]. This framework as conceptualised by Levesque, Harris et al (2013) provide four interrelated domains for explaining the access pathway to healthcare by communities. These include geographical, affordability accommodation and acceptability. The questionnaire was divided into four sub-sections corresponding to these four adapted dimensions of the health access. For each dimension contained between 5 to 16 Likert-scale (scale of 0-4) questionnaire items developed to cover the concepts central to that healthcare

For peer review only - http://bmjopen.bmj.com/site/about/guidelines.xhtml

access dimension in the context of cross-border settings. This method is built around exploratory factor analysis and it is recommended for assessing multi-dimensional concepts such as healthcare access in urban setting [24,25]. The questionnaire items for each sub-dimension were phrased to reflect the pathway of the client's decision to seek care across the border up to the time of receiving care or failing to receive care. Each sub-dimension also had an overall Likert-scale item to assess overall contribution of that dimension to their recent experience of cross-border healthcare access.

The questionnaire had one open-ended question that asked each of the study respondents to give a narration of their most recent vivid experiences[26] seeking health care across the border. During the narration, we probed for factors that enabled navigation of barriers for example: cost of care, crossing requirements and quality of care. The narrations were audio recorded with consent of the respondents and transcribed verbatim.

The questionnaire content validation was explored by two rounds of workshops by the research team. The designed tool was pretested at one similar site in Uganda before initiating data collection. Analysis of pre-test data showed adequate (above 0.70) Cronbach's alpha reliability coefficients for the items in all dimensions of the tool after elimination of few questionnaire items. The tools were translated into the local language of the respective data collection site. The languages used included: Swahili (Kenya), Kinyarwanda (Rwanda), Rukiga (Uganda-Katuna border) and Lusyamya (Uganda-Busia border). As part of the translation process, the tool was translated from the original English version into the site-specific local language (Swahili, Kinyarwanda, Rukiga and Lusyamya). Back translation from local language versions back to English was used compared with the original version[27]. The interviews were collected by a team of experienced and well-trained young researchers on the study team.

Data management and analysis

Quantitative data management and analysis

Filled questionnaires were checked for completeness, assigned a unique identifier and entered in *Epi Data*. The summary data set was then exported to *SPSS Version 23* for analysis. In preliminary analysis, we determined the reliability of the items in each of the 4 sub-sections of the questionnaire separately using the Cronbach's alpha reliability coefficient

Next, we conducted dimension reduction using Exploratory Factor Analysis (EFA) in *SPSS version 23* to generate fewer and meaningful (latent) variables with eigenvalues of 1 and above.

For peer review only

To retain variables that clearly discriminate between the generated latent variables, the minimum cut off for item loading was set at 0.7.

Finally, a regression was performed to determine the independent association of each of the generated latent variables to the dependent variable - “perceived ease of access to health care” on the opposite side of the border. Beta co-efficient, Confidence Interval (CI) and P values were used to gauge the strength of association of latent factors with the dependent variable. Results were stratified by case condition and by dimension of the health access framework.

Qualitative data management and analysis

For the qualitative data, the recordings and all handwritten notes were kept in a secure location at the study secretariat office of the partnering universities that led the data collection. No identifying information of any of the specific respondents was included in the transcription and analysis reports. Qualitative data were exported to *Atlas TI version 8* for analysis. A conventional deductive content analysis approach was used [28]. From the text, codes were derived then sorted into categories then grouped together to inform emerging overarching themes. The analysis took into considerations any relevant comparisons in the different study sites, country where health care was sought, gender and case condition. Relevant qualitative quotes from respondents were presented in the results sections to support the key results under the respective themes and categories. In order to ensure trustworthiness, each transcript was coded by two researchers and any differences were discussed by the research teams to reach consensus. The derivation of the sub-themes and themes was done in a workshop with all the authors. The research team has a good mix of both qualitative and quantitative expertise.

Ethical considerations

The study was approved by the institutional review boards of the participating universities and the national research regulation councils in Uganda, Kenya, Tanzania and Rwanda were study activities were implemented. The community survey was not done in Tanzania due to unanticipated changes in the study team there and logistical challenges related to travel. All study respondents provided informed consent prior to participating in the study.

Patient and public involvement

Prior to the data collection field work, the study team conducted a study pre-visit meeting to all the study sites and engaged local leaders, health officials and border managers in order to introduce the study objectives and seek their permission and consent to participate in the study.

For peer review only - http://bmjopen.bmj.com/site/about/guidelines.xhtml

The study team worked with some of the community members as field guides during the data collection. The study team held dissemination meetings at all the study sites in order to validate the findings.

Results

<u>*Respondent characteristics*</u>

A total of 279 households were surveyed. The majority of respondents were female (n=251, 90%), and married (n=213, 76.3%). Only 4 percent (n=11) were in formal employment. The majority had relatives across the border in the neighboring country 75% (n=207). The detailed characteristics of the respondents are summarized in table 1.

[Table1: Socio-demographic characteristics of the household adults who reported a household member who had ever crossed the border to seek healthcare services]

<u>*Characteristics of individuals who accessed health-care from the opposite side of the border*</u>

Majority of the people who had accessed health care from the opposite side of the border were female (n=205, 73.5%). Those accessing care for more than a year ago were 27.6% (77/279) while 22.9% (64.279) had accessed care in less than a month prior to interview. The majority accessed care from public health facilities (n=234, 88.9%), while 104 (37.3%) had sought immunisation services. The details are summarized in table 2.

[Table 2: Characteristics of border residents who accessed health care from the opposite side of the border]

Reliability analysis of the survey questionnaire

The respondents were surveyed on four (4) dimensions of cross border health-care access. The reliability analysis of the survey questionnaire, reported as Cronbach's alpha showed the following results. Physical; 0.647, Affordability; 0.215, Availability and Accommodation; 0.783, Acceptability and Appropriateness; 0.645

<u>*The most significant factors that facilitate successful access to health-care from the opposite side of the border*</u>

Principal component analysis with varimax rotation was conducted in SPSS to generate latent variables for all the access dimensions. The eigenvalues greater than one rule and scree tests were used to decide the numbers of factors/latent variables to retain. The latent variables were

For peer review only - http://bmjopen.bmj.com/site/about/guidelines.xhtml

labelled according to the items that loaded onto them the most[29]. For example, six new/latent variables were generated for the physical access dimension. The items that loaded onto the second latent variable in the physical access dimension are items C14 and C16. To decide the name of this new variable, we merged the meaning of C14 and C16 and called the variable "Social Connections''. This was done for all the latent variables across the four (4) access dimensions. These findings are summarized in figure 2.

[Figure 2 caption: New generated variables for each of the 4 studied access dimensions and corresponding items that loaded with a factor of 0.7 and above onto the new variables]

Overall ease of cross border access to healthcare

The Likert-scale median scores ranged from 3-4. Overall, all access dimensions were important factors in successful cross border health access. For different case conditions however, different access dimensions had different levels of contribution to successful cross border health access. For respondents who accessed maternal delivery services across the border, availability and accommodation dimension contributed more to their successful cross border healthcare access. For respondents who accessed HIV treatment services, affordability contributed more. Service related findings are summarized in figure 3.

[Figure 3 caption: The extent to which each access dimension contributed to the ease of accessing healthcare from the opposite side of the border for each of the 3 case conditions]

Independent predictors of cross border health access

Separate regressions were run against the mean score of the outcome variable "overall ease of access of cross border health care" for all the access dimensions. In all regressions, the independent variables were the latent variables extracted using factors analysis. The regression analysis was also stratified for each of the 3 case conditions – HIV treatment, childhood immunization and maternal delivery.

For physical access, the model explained 36% variation, which varied between 38-40% depending on the stratification by the three case conditions. The strongest independent predictor of ''overall ease of physical crossing for care'' was ''ease of border crossing''. For financial access/affordability, the model explained 31% variation, which varied between 21-44% depending on the case condition. Access to free care was (inverse of Self-funding) was one of the main predictors. Table 3 provides the main results.

[Table 3: Independent predictors of overall ease of cross border access to health care]

For peer review only - http://bmjopen.bmj.com/site/about/guidelines.xhtml

Qualitative findings:

From the qualitative analysis, two overarching themes emerged and these were reasons for crossing the border to seek health care and experiences on how resident communities navigate barriers during cross border healthcare access. The results are summarized in table 1 in supplementary file 1 attached

Reasons for crossing the border to seek health care

The main push factors for crossing to seek care across the border were: 1) service delivery points were more accessible, 2) health services were more affordable, 3) availability of services needed, and 4) acceptability of the services

Physical accessibility of the health services

The border residents preferred to access services that are closer to them. In instances where the nearest health facilities was on the opposite side of the border, the residents preferred to cross the border to access services rather than travelling long distances to access services in their own country;

> *"The reason I went to Uganda, it is because the health facilities where we get our vaccination (RUBAYA, CYUMBA and KANIGA) are more distant, and the transport to reach there is expensive"* - **Border resident, Gatuna-Rwanda.**

Having relatives and friends on the opposite side of the border was mentioned as a reason why most border residents preferred to seek care across the border since they had an opportunity of being taken care of by their relatives:

> *"My relative live there, so I went there because they could take care of me after giving birth …. I just crossed because I knew my family would receive me"*-**Border resident, Gatuna-Rwanda.**

Affordability of the health services

Health services being more affordable prompted study communities to seek healthcare by crossing the border. This was more common where national health insurance was established in the home country and someone wanted to access services that are not covered by insurance at home. They crossed to where there was a "free services" policy. They further noted that accessing these services without insurance in their own country was more costly. One respondent noted:

> *"It all started when I was coming from the hospital … I needed medication to recover, but, the medicines I required, were not covered under my health insurance. So a friend of mine recommend me to go to Uganda side"*- **Border resident, Gatuna-Rwanda**.

For peer review only - http://bmjopen.bmj.com/site/about/guidelines.xhtml

For peer review only

Availability of the health services

The inadequacies of services in one's home country often prompted border resident communities to cross. Border residents who visited the health facilities in their home countries and did not get the services they required tended to cross the border to access these services;

> *"I took my grandchild because after visiting the hospitals here for three consecutive times and missing the vaccine, I was forced to travel to Uganda for the vaccine"*- **Border Resident, Busia-Kenya.**

Formal and informal referral to quality, nearer and cheaper health facilities prompted border residents to seek healthcare from health facilities in the opposite side of the border. Formal referrals, were arranged by a health worker while informal referrals were prompted by self, friends, relatives and other community members.

> *"I started feeling labor pains where I visited the district hospital but got students who were attending to patients. I was bleeding and was advised to go to MASAFU (Uganda side) for delivery"*- **Border resident, Busia-Kenya.**

Acceptability of health services

This theme was related to the confidentiality of care sought across the border and perceived better quality of the care across the border. The fear of being stigmatised by fellow community members was also found among a few respondents. This prompted HIV positive border residents to seek health care from health facilities across the border fearing that home communities would come to know about their HIV sero-status;

> *"When I discovered I was infected with HIV I was stigmatized and couldn't stand going to pick drugs as I could meet very many people I know. I was forced to go to Uganda and gained courage when picking the drugs"*-**Border resident, Busia-Kenya.**

Border residents who perceived that services from the opposite side of the border were of better quality than at home tried to seek healthcare from health facilities across the border;

> *"When I went to the Kenyan hospital they worked on me well and I came back safely. The good thing in Kenya is that any nurse can help you, she tells you where to start from, and she directs where to get the book and notes all things concerning the baby."*- **Border resident, Busia-Uganda**

Experiences on how border resident communities navigated barriers during cross border health care access

From the narration of experiences with household adults that had successfully sought care across the border, the key facilitators that enabled navigation of barriers emerged across the physical and affordability dimensions. Under physical dimension, the main facilitators were

For peer review only

presence of informal routes, having official travel documents and knowing a similar language. While, under affordability the main facilitator was the ability to meet the cost of care across the border.

Presence of informal routes

The presence of informal routes locally known as *"panya roads"* facilitated access to health care services from the opposite side of the border. Many border resident communities were not allowed to cross at the formal crossing routes for health services and resorted to using informal routes. We also found a common ethnic identify of communities separated by the national borders. These communities speak the same local language across the border, they use both formal and informal routes to cross the border for health, markets and other reasons. Many community members have informal connections or relatives across borders but also are capable of independent and informal navigation of the healthcare access that bypass formal border controls and protocols.

> *"When I pass via the customs offices, they sometimes ask for money or arrest me but I was arrested once. Now, I normally pass via a panya (short cut route) (to Kenya side) to pick my medicine"* - **Border resident, Busia-Uganda.**

> *"Border officers send you back because all those services are available here….(in Rwanda). Actually, most people who need those services use shortcuts to cross. Furthermore, Ugandans who take HIV treatment here (in Rwanda) also pass through there (the informal route)"* – **Border resident, Gatuna- Rwanda**

Having official travel documents

Border residents who had their identification documents, such as; identity cards and passports were easily allowed to cross the border. Most of the border residents without travel documents were not allowed to cross the border and, in most instance, they had to make informal payments or bribes so as to be allowed to cross. Some respondents however mentioned that in some instances, travel documents were not a requirement for crossing the border and this facilitated health care access. One respondent remarked:

> *"No, whenever I visit there I am never asked for any document either when going or coming back*"- **Border Resident, Busia-Kenya.**

Similarity in language

Knowing the local dialect of the neighbouring border communities facilitated access to health care services across the border. Border residents were able to easily communicate with the service providers and even to disguise as nationals of the destination country. On the other

For peer review only - http://bmjopen.bmj.com/site/about/guidelines.xhtml

hand, the inability to speak the local language hindered access to health care services due to language barrier and sometimes had to get translators.

> "*I was just treated well…It's only the language barrier that made the whole process look tiresome*"**-Border Resident, Isebania-Kenya**.

Affordability of cost of care

The cost of care included the money paid for the actual health services, the transport costs and other informal payments or bribes made to facilitate the process of accessing care. The respondents who had crossed to access care on the opposite side of the border unanimously agreed that their ability to pay the actual cost of services enables them to access health services from there. In most cases, HIV treatment and childhood immunisation services were free and these enabled border residents with these conditions to access care.

> *"The cost was so much reasonable as compared to Kenyans side and we also used Kenyan currency which is stronger that the Uganda shillings"*- **Border Resident, Busia-Kenya.**

Relatedly, being able to pay informal payments/ bribes either at the border point (to border officials) or at the health facility enabled access to health services at the opposite border

> *"While on the way we encountered the police whom we bribed to proceed with our journey to Dabani Hospital. At the hospital we had a midwife who was attending to her by bringing her food and water to bath and we ended up paying her (the midwife)"***- Border Resident, Busia-Kenya.**

Discussion

This study aimed to explore how to increase health care access for communities along the border in East Africa. We found that communities that are resident near national borders were able to cross borders to seek health care. There are factors that attract border residents to seek care across the border and these centred on service delivery points being near, availability of services and health services being of acceptable quality across the border compared to the home country. From the quantitative analysis the main facilitators for cross border health access were ease of physically crossing the border and health services being affordable across the border among other factors. In general, the qualitative and quantitative results were in agreement and these are discussed together below.

Rather than travelling long distances to access healthcare in the home country, border residents preferred to access services that were closer to them though across the border. This has also

For peer review only - http://bmjopen.bmj.com/site/about/guidelines.xhtml

been seen elsewhere [30] that patients gravitate to their nearest convenient health service provider. Other than physical "closeness" of the health care across the border, many border residents in our study commonly used informal routes "*Panya roads*" to cross for health care – an indication of porous borders referred to as "ease of border crossing" as seen in the liner regression analysis where "ease of border crossing" was an important factor that enabled health access across the border. The ease of physical access is also enhanced by the fact that for many of the border communities, the culture and language is the same and they live as one community irrespective of the border [17]. This is evidenced from the respondents' remarks that knowledge of the local language and family relatives that reside across the border facilitated crossing for health care.

Border residents chose to seek health care across the border because it was still affordable compared to care within their home country. This is in concordance with the linear regression analysis where the most important factor in the affordability dimension was the inverse of "self-funded" since most services are usually free especially for the case conditions explored in our study. Studies done among U.S. residents living along the U.S.–Mexico border have shown that due to the economic, financial, and health care access barriers, border residents on the US side commonly resort to crossing into Mexico to meet their health care needs because of much more affordable prescription medications as well as services [6,7]. For the border residents in East Africa, there seemed to be an opportunity cost they were willing to pay in terms of other costs like transport and informal payments at border crossing. However, we acknowledge that this opportunity cost was easier for the border residents to bear since they were seeking services like childhood immunization and HIV treatment refill which are usually free of charge in East Africa [31]. Apart from affordability in terms of cost of care, border residents who lacked health insurance also preferred to seek care across the border where national health insurance was not being implemented. There is need for East African countries to have common arrangements for example health insurance with portable benefits (or free care) that especially help poor communities along the border to access care. Although currently the non-insured community members can access care across the border, this leaves them disadvantaged in case the service needed is only available in their home country [32].

Some border residents also preferred to go where care was perceived to be acceptable in terms of quality and confidentiality. This was more important for mothers seeking delivery services and HIV patients that preferred confidentiality. This speaks to the need for countries to

For peer review only - http://bmjopen.bmj.com/site/about/guidelines.xhtml

improves service quality so that citizens in remote areas are more willing to use the available services at home [33].

From our study, we see the need to move from nationally circumscribed rights and entitlements to a legal environment that allows border residents to freely access care cross the border if the nearest service delivery point is on the opposite side of the border within a well-established and agreed upon arrangement between countries. Though border residents are individually navigating access barriers, this leaves them vulnerable if some legal frameworks were to be enforced. For UHC to be efficiently delivered, countries may need to agree upon and establish planning, resourcing and regulatory arrangements to enable border communities access the existing healthcare provision without bearing the hardship of navigating these barriers on their own [22]. This can also reduce the uncertainty and discretion of front-line officials if they were all aware of the access rights for border residents in neighboring communities. This can span to other cross border health issues such as having joint efforts to deal with disease outbreaks[34,35].

At regional level, centers of excellence are beginning to form with a view of serving people from the EAC [36] and there is need for innovative arrangement at micro level for efficient delivery of health care. It would be useful to share the available healthcare resources (for example, every side of the border does not have to build a hospital if one side of the border has one in close distance). Guidelines should be developed to enable the use of a common pool of health care resources and optimize efficiency on both sides of the border. The European region has tried to remove legal and administrative barriers through cross-border cooperation (CBC) [37] aimed at "identifying potential complementarities in all fields of human activities in the border regions to ease the lives of the border residents". There are lessons that can be driven from these and other cross-border integration initiatives [13]. During the design of any cross border cooperation initiatives, host communities should be engaged in the plans as part of the efforts to promote "tolerance, understanding and good neighbourly relations between populations" [38]

As this paper goes to publication, cross border health has been made more urgent by the Corona virus pandemic and its impact on the border health systems to provide testing and surveillance for interstate truck drivers in East Africa. The guidelines to manage the pandemic have come into sharp confrontation with the objectives for legal rights of interstate travel and movement

For peer review only - http://bmjopen.bmj.com/site/about/guidelines.xhtml

of goods – in some cases threatening to dismantle the regional cooperation mission [39]. Numerous media carried stories showing long queues of trucks some extending 20 Km at the border all waiting for Corona virus testing that was sometimes taking 2-3 days to offer the test results and clear the truck through emigration points. Communities around these long jams of trucks were also exposed to community transmission of coronavirus from the truck drivers – a group that had a high occupational risk of contracting and transmitting the virus [40]. This further demonstrates the need to invest in and coordinate better the economic and health care objectives at border points for the East African community to consolidate its integration and social economic functionality.

Study limitations:

This paper only presents the views of respondents that successfully navigated access barriers. Our study sites were the official cross points. We did not include the numerous informal cross points which may have different contexts and experiences. The use of official crossing was selected to enable the study to factor in the operations of the formal rules for emigration when seeking health care across the border.

Conclusion

From this study, we note that communities resident near national borders of the EAC were able to cross borders to seek health care. Although the EAC integration agenda has been discussed for a while, progress has been made in the areas of economics and trade and it has not yet benefited border residents in terms of access to healthcare across the border. There is need for: innovative arrangements to optimize the existing resources and a policy environment to enable East Africa invest better and realize synergies for these communities for health integration in the EAC.

Acknowledgments

The authors thank the District or County Leadership at the following border sites: Katuna-Uganda (Kabale District); Gatuna-Rwanda (Gicumbi District); Rusomo-Rwanda (Kibehe District); Busia-Uganda (Busia District); Busia-Kenya (Busia County); Isebania-Kenya (Migori County). Thanks to Makerere University School of Public Health, SPEED Project and many thanks to the following persons for their key role in the data collection activities: Mr Moses Sitati, Researcher at Moi University who led the field team in Kenya, Shadat Kititi of

For peer review only

Makerere University School of Public Health who led the field team in Uganda and Vincent Mutabazi of University of Rwanda who led the field team in Rwanda.

Contributors

FS, DT, SS, SB, AS, LM and MN conceptualised the study. FS, DT and SB led the team's field work. FS, LM and MN sought country level study approvals. FS, DT, SB, SS, AS, MA, AR, LM and MN led the study pre-visits and data collection. FS, SS, MA and AS led the quantitative data analysis while FS, DT, SB, AR, LM and MN led the qualitative data analysis. FS, DT, SS and MA wrote the draft manuscript, with all authors providing feedback and approving the final version

Ethics approval and consent to participate: Ethical approval for this study was provided by Makerere University Higher Degree Research and Ethics Committee (HDREC Protocol number 583) and the Uganda National Council of Science and Technology (Reg No SS4713). In Kenya the study was approved by Moi University/Moi Teaching Referral Hospital Research and Ethics Committee (FAN: IREC: 3077); in Rwanda was approved by the Rwanda National Ethics Committee (No.438/RNEC/2018) and in Tanzania by the University of Dar es Salaam Research Ethics Committee (Reference Number UDSM-REC/2018/02). All study respondents provided informed consent prior to participating in the study.

Consent for publication: Not applicable.

Availability of data and materials: The datasets used and/or analyzed during for our study are available from the corresponding author upon reasonable request.

Competing interests: The authors declare that they have no competing interests

Funding: This work was supported by MRC/Wellcome Trust, UK, grant number: MR/R02028/1

Figure captions

For peer review only - http://bmjopen.bmj.com/site/about/guidelines.xhtml

574 **Figure 1**: Study sites (indicated by red circles

575 **Figure 2**: New generated variables for each of the 4 studied access dimensions and corresponding items that loaded with a factor of 0.7 and above onto the new variables

577 **Figure 3**: The extent to which each access dimension contributed to the ease of accessing healthcare from the opposite side of the border for each of the 3 case conditions

For peer review only

References

1. Mansourian BP. *Global Perspectives in Health-Volume II.* Vol 3: EOLSS Publications; 2009.

2. Rodríguez-Saldaña J. Challenges and opportunities in border health. *Preventing chronic disease.* 2005;2(1).

3. United States Mexico Border Health Commission. Border lives: health status in the United States-Mexico border region. *El Paso (TX): United States–Mexico Border Commission.* 2010.

4. Legido-Quigley H, Glinos I, Baeten R, McKee M. Patient mobility in the European Union. *Bmj.* 2007;334(7586):188-190.

5. Ried W, Marschall P. Potential benefits of crossborder health care: The case of Poland and Germany. *Economics & Sociology.* 2016;9(3):14.

6. Miller-Thayer J. Health migration: crossing borders for affordable health care. *Field Actions Science Reports. The journal of field actions.* 2010(Special Issue 2).

7. Su D, Richardson C, Wen M, Pagán JA. Cross-border utilization of health care: evidence from a population-based study in south Texas. *Health services research.* 2011;46(3):859-876.

8. Levesque J-F, Harris MF, Russell G. Patient-centred access to health care: conceptualising access at the interface of health systems and populations. *International journal for equity in health.* 2013;12(1):18.

9. Hanson K, Ranson MK, Oliveira-Cruz V, Mills A. Expanding access to priority health interventions: a framework for understanding the constraints to scaling-up. *Journal of International Development: The Journal of the Development Studies Association.* 2003;15(1):1-14.

10. Andersen R, JF N. Andersen and Newman framework of health services utilization. *Journal of Health and Social Behavior.* 1995;36:1-10.

11. Bruhn JG. *Culture and health disparities: evaluation of interventions and outcomes in the US-Mexico border region.* Springer; 2014.

12. Hunter-Adams J, Rother H-A. A Qualitative study of language barriers between South African health care providers and cross-border migrants. *BMC Health Services Research.* 2017;17(1):97.

13. Böhm H, Kurowska-Pysz J. Can Cross-Border Healthcare Be Sustainable? An Example from the Czech-Austrian Borderland. *Sustainability.* 2019;11(24):6980.

14. OHCHR and WHO. *The Right to Health. Fact sheet no. 31.* Office of the United Nations High Commissioner for Human Rights (OHCHR) and The World Health Organization (WHO);2008.

For peer review only

For peer review only - http://bmjopen.bmj.com/site/about/guidelines.xhtml

616 15. Derose KP, Gresenz CR, Ringel JS. Understanding disparities in health care access—and reducing them—through a focus on public health. *Health Affairs.* 2011;30(10):1844-1851.

619 16. Van Reisen M, Mawere M, Stokmans M, Gebre-Egziabher KA. *Roaming Africa: Migration, Resilience and Social Protection.* Vol 2: Langaa RPCIG; 2019.

621 17. Durham J, Blondell SJ. A realist synthesis of cross-border patient movement from low and middle income countries to similar or higher income countries. *Global Health.* 2017;13(1):68-68.

624 18. Leontidou L, Afouxenidis A, Donnan H, et al. Border Cities and Towns: Causes of Social Exclusion in Peripheral Europe. Brussels: European Commission. Targeted Socio-Economic Research (TSER) SOE2 …; 2001.

627 19. Maessen JH, Raab J, Haverkate M, et al. How prepared are we for cross-border outbreaks? An exploratory analysis of cross-border response networks for outbreaks of multidrug resistant microorganisms in the Netherlands and Germany. *PloS one.* 2019;14(7).

631 20. Nsoedo EE. The Ebola Crisis in the West African region: should it have been so severe? *Open Journal of Social Sciences.* 2014;2(10):98.

633 21. Wetzel D. Pandemics know no borders: In Africa, regional collaboration is key to fighting COVID-19

635 2020; https://blogs.worldbank.org/africacan/pandemics-know-no-borders-africa-regional-collaboration-key-fighting-covid-19. Accessed 24th June 2020.

637 22. UN. *Transforming Our World: The 2030 Agenda for Sustainable Development.* United Nations;2015.

639 23. Adams W. Conducting Semi-Structured Interviews. In: Kathryn E. Newcomer HPH, Joseph S. Wholey, ed. *Handbook of practical program evaluation*: John Wiley & Sons; 2015.

642 24. Gaskin CJ, Happell B. On exploratory factor analysis: A review of recent evidence, an assessment of current practice, and recommendations for future use. *International journal of nursing studies.* 2014;51(3):511-521.

645 25. McDade TW, Adair LS. Defining the "urban" in urbanization and health: a factor analysis approach. *Social science & medicine.* 2001;53(1):55-70.

647 26. deMarrais KB, Lapan SD. *Foundations for research: Methods of inquiry in education and the social sciences.* Routledge; 2003.

649 27. Lee J-AL, More SJ, Cotiw-an BS. Problems translating a questionnaire in a cross-cultural setting. *Preventive veterinary medicine.* 1999;41(2-3):187-194.

651 28. Hsieh H-F, Shannon SE. Three approaches to qualitative content analysis. *Qualitative health research.* 2005;15(9):1277-1288.

For peer review only - http://bmjopen.bmj.com/site/about/guidelines.xhtml

29. Costello AB, Osborne J. Best practices in exploratory factor analysis: Four recommendations for getting the most from your analysis. *Practical assessment, research, and evaluation.* 2005;10(1):7.

30. Welsh Affairs Committee. The Provision of Cross-border Health Services for Wales. *London, The Stationery Office,(HC 56 of 2008–09).* 2008.

31. MEASURE Evaluation. *East Africa Cross Border Integrated Health Study.* The Carolina Population Center, University of North Carolina 2017. 978-1-9433-6469-5.

32. Busse R. Border-crossing patients in the EU. *EUROHEALTH-LONDON-.* 2002;8(4):19-21.

33. Suñol R, Garel P, Jacquerye A. Cross-border care and healthcare quality improvement in Europe: the MARQuIS research project. *Qual Saf Health Care.* 2009;18 Suppl 1(Suppl_1):i3-i7.

34. WHOAFRO. Ten African countries endorse cross-border collaboration framework on Ebola outbreak preparedness and response. 2019; https://www.afro.who.int/news/ten-african-countries-endorse-cross-border-collaboration-framework-ebola-outbreak-preparedness. Accessed 16th May, 2020.

35. Olu OO, Lako R, Wamala JF, et al. What did we learn from preparing for cross-border transmission of Ebola virus disease into a complex humanitarian setting – The Republic of South Sudan? *Infectious Diseases of Poverty.* 2020;9(1):40.

36. EASTECO. *Concept Note: THE SECOND EAC REGIONAL E-HEALTH & TELEMEDICINE WORKSHOP, MINISTERIAL CONFERENCE AND TRADE EXHIBITION, Kigali, Rwanda, 15-18 May 2018.* The East African Science and Technology Commission;2018.

37. EC. *Easing legal and administrative obstacles in EU border regions.* Luxembourg: European Commission;2017.

38. Council of Europe. *Cross-border Co-operation Toolkit.* Centre of Expertise for Local Government Reform, Council of Europe;2012.

39. Victor B. Stop Criminalising Truck Drivers In War Against COVID-19. 2020; 13th June 2020. Available at: https://www.capitalfm.co.ke/news/2020/05/stop-criminalising-truck-drivers-in-war-against-covid-19/, 2020.

40. Address by H.E. Yoweri Kaguta Museveni, President of the Republic of Uganda to the nation: Latest Updates on Matters Regarding Corona Virus (COVID 19) [press release]. Kampala: The State House of Uganda, 4th May 2020 2020.

For peer review only

For peer review only - http://bmjopen.bmj.com/site/about/guidelines.xhtml

Table 1: Socio-demographic characteristics of the household adults who reported a household member who had ever crossed the border to seek healthcare services

Characteristic	Frequency (n)	Percentages (%)
Sex		
Male	28	10.0
Female	251	90.0
Age (Mean, SD)	Mean = 32.8	SD = 9.8
Educational Level		
No Education	35	12.5
Some Primary	107	38.4
Completed Primary	48	17.2
Some Secondary	49	17.6
Completed Secondary	27	9.7
Tertiary	13	4.7
Marital Status		
Single	28	10.0
Married	213	76.3
Divorced	16	9.3
Widowed	12	4.3
Employment Status		
Not Employed	131	47.3
Informal & Self Employment	135	48.7
Formal employment	11	4.0
Nationality		
Kenyan	82	31.1
Rwandese	89	33.7
Tanzanian	6	2.3
Ugandan	86	32.6
Dual Citizenship	1	0.4
Has relatives on opposite border		
Yes	207	75.0
No	69	25.0
Border site		
Busia Kenya-Busia Uganda	97	34.8
Gatuna-Katuna	91	29.0
Isebania	49	17.6
Rusumu	52	18.6
Country of residence		
Kenya	84	30.1
Rwanda	101	36.2
Uganda	94	33.7

For peer review only

Table 2: Characteristics of border residents who accessed health care from the opposite side of the border

Characteristic	Frequency (n)	Percentages (%)
Age (Mean, SD)	Mean = 22.7	SD = 16.7
Sex		
Male	74	26.5
Female	205	73.5
When care was sought		
< 1 Month	64	22.9
1 to 3 Months	58	20.7
>3 to 6 Months	26	9.3
>6 to 12 Months	54	19.3
>12 Months to 5 years	77	27.6
Type of facility where care was sought		
Public	234	83.9
Private Not For Profit	11	3.9
Private For Profit	32	11.5
Don't Remember	2	0.7
Service received		
Delivery	98	35.1
HIV treatment	77	27.6
Immunisation	104	37.3
Type of service received		
Diagnosis Only	3	1.1
Treatment as OPD	192	68.8
Admission	40	14.3
Operation	44	15.8
Reasons why care was sought		
Better quality	99	36.0
Referral	10	3.6
Less Costly	79	28.7
Community Support	18	6.6
Stigma	12	4.3
Proximity	25	9.0
Other	51	18.6

For peer review only

Table 3: Independent predictors of overall ease of cross border access to health care

Access dimension	**Regression model items**	**Overall**		**Delivery**		**HIV**		**Immunization**	
		Regression coefficient	P-Value	Regression coefficient	P-Value	Regression coefficient	P-Value	Regression coefficient	P-Value
Physical Access	Constant	3.221	0.000	3.23	0.000	3.15	0.000	3.254	0.000
	Ease of border crossing	**0.381**	**0.000**	**0.346**	**0.000**	**0.565**	**0.000**	**0.371**	**0.000**
	Social connections	-0.79	0.061	-0.09	0.209	-0.035	0.702	-0.05	0.475
	Satisfaction with organization and approachability of facilities at home	-0.006	0.883	-0.012	0.865	0.126	0.130	-0.105	0.138
	Documentation of referral for health care needs	**0.098**	0.021	0.146	0.085	0.034	0.627	0.133	0.066
	Satisfaction with the organization and approachability of facilities across the border	**0.156**	**0.00**	**0.164**	**0.037**	0.123	**0.140**	**0.217**	**0.002**
	Distance to facility	**0.224**	**0.00**	**0.213**	**0.004**	**0.316**	**0.002**	**0.226**	**0.001**
	R square	0.361		0.379		0.402		0.388	
	N	251		89		69		91	
Financial access/ Affordability	Constant	3.009	0.000	2.918	0.000	2.78	0.000	3.027	0.000
	Self-funded	**-0.478**	**0.000**	**-0.496**	**0.000**	**-0.646**	**0.000**	**-0.387**	**0.000**
	Time & transport cost acceptable	0.004	0.946	-0.075	**0.344**	**0.488**	0.002	-0.049	0.624
	Cost of care affordable	**0.324**	**0.00**	**0.522**	0.000	0.147	0.126	**0.260**	**0.013**
	Insurance needed	0.006	0.917	0.018	**0.821**	**-0.233**	0.041	0.154	0.133
	Local currency and financial support	-0.047	0.406	-1.13	0.151	0.066	0.525	-0.049	0.616
	R square	0.305		0.437		0.437		0.213	
	N	248		95		62		89	
Availability/ Accommodation	Constant	3.451	0.000	3.443	0.000	3.487	0.000	3.411	0.000
	Better workforce	**0.106**	**0.002**	0.045	0.461	**0.216**	0.001	0.108	0.075
	Provider Public-Private mix	0.064	0.063	0.047	0.442	0.002	0.978	0.103	0.114
	Services/medicines available	**0.274**	**0.00**	**0.297**	**0.000**	**0.393**	**0.000**	**0.144**	**0.040**
	Not getting better at home	0.04	0.242	0.023	0.723	0.063	0.224	0.033	0.580
	Language was not a barrier	0.021	0.549	0.011	0.850	-0.01	0.864	0.061	0.349
	R square	0.242		0.295		0.471		0.113	
	N	252		90		70		90	
Acceptability and Appropriateness	Constant	3.395	0.000	3.37	0.000	3.311	0.000	3.462	0.000
	Acceptable Quality	**0.364**	**0.000**	**0.388**	**0.000**	**0.372**	**0.000**	**0.325**	**0.000**
	Accountability	**0.142**	**0.000**	**0.154**	**0.004**	**0.228**	**0.000**	0.062	0.304
	R square	0.364		0.462		0.396		0.246	
	N	262		92		73		95	

For peer review only - http://bmjopen.bmj.com/site/about/guidelines.xhtml

Figure 1: Study sites (indicated by red circles)

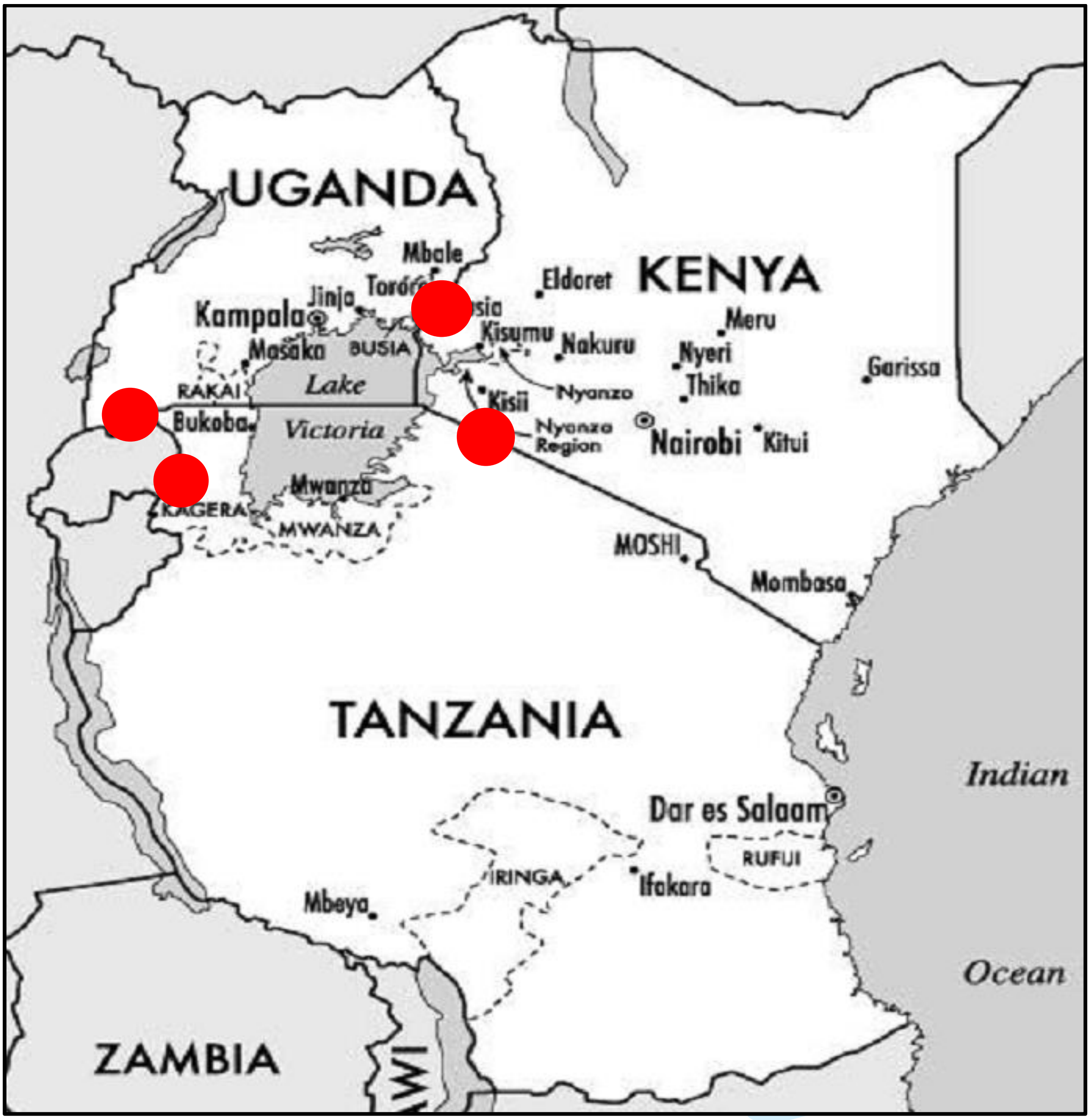

Figure 2: New generated variables for each of the 4 studied access dimensions and corresponding items that loaded with a factor of 0.7 and above onto the new variables

Physical access Dimension

Items	Latent variables/Item loadings					
	Ease of crossing the border	Social Connections	Satisfaction with organization of service at home	Referral Documents	Satisfaction with organization of services across the border	Distance to the health facility
c1						.860
c2	.702					
c3	.756					
c5			.873			
c6			.905			
c7					.857	
c8					.822	
c10	.728					
c12				.751		
c13				.739		
c14		.770				
c16		.707				

Financial access/ Affordability Dimension

Items	Latent variables/Item loadings				
	Self-funded	Time & transport cost acceptable	Cost of care affordable	Insurance needed	Local currency and financial support
d1			.745		
d3		.875			
d4		.832			
d5	.862				
d7	-.824				
d9					.793
d10	.825				
d11				.826	

Availability and Accommodation Dimension

	Latent variables/Item loadings				
	Better Workforce	Provider Public-private mix Across	Services & medicines available Across	Not getting better at home	Language was not a barrier
e1				0.797	
e2				0.717	
e4			0.842		
e5	0.855				
e6	0.825				
e8			0.791		
e10					
e11		0.709			
e13		0.718			
e16					0.835

Acceptability and Appropriateness Dimension

	Latent variables/Item loadings	
Items	Acceptable Quality	Accountability
f1	.900	
f2	.911	
f3		
f4		.858
f5		.811

Figure 3: The extent to which each access dimension contributed to the ease of accessing healthcare from the opposite side of the border for each of the 3 case conditions

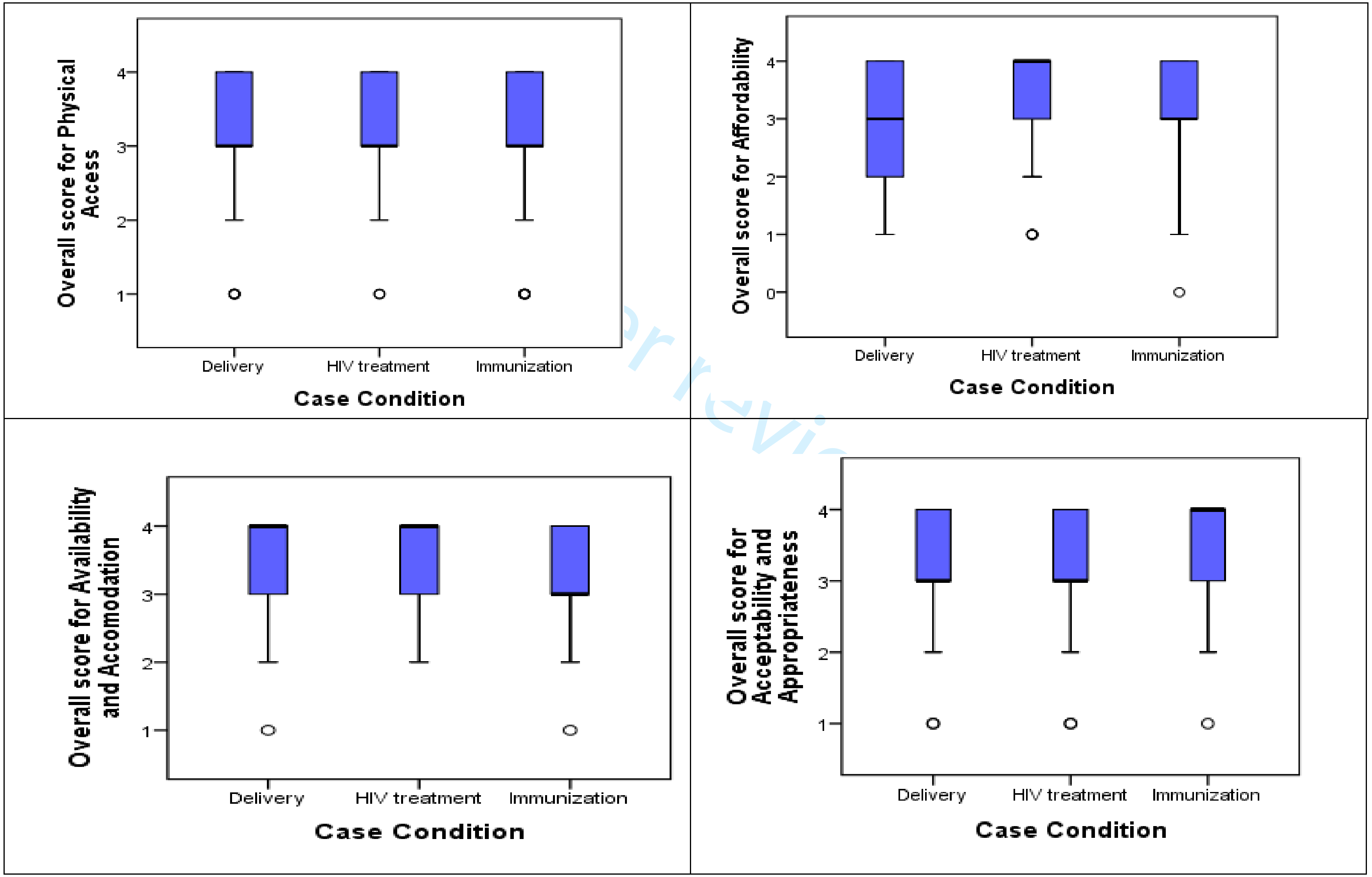

Supplementary Table 1: Summary qualitative themes and sub-themes.

Reasons for crossing the border to seek health care		
Theme	Sub themes	Illustrative quote
****Physical accessibility of the health services	Nearest health facilities is on the opposite side of the border	*"The reason I went to Uganda, it is because the health facilities where we get our vaccination (RUBAYA, CYUMBA and KANIGA) are more distant, and the transport to reach there is expensive"* - Border resident, Gatuna-Rwanda.
	Having relatives and friends on the opposite side of the border	*"My relative live there, so I went there because they could take care of me after giving birth …. I just crossed because I knew my family would receive me"*-Border resident, Gatuna-Rwanda.
***Affordability of the health services	Health services more affordable across the border	*"It all started when I was coming from the hospital … I needed medication to recover, but, the medicines I required, were not covered under my health insurance. So a friend of mine recommend me to go to Uganda side"*- Border resident, Gatuna-Rwanda.
**Availability of the health services	Presence of needed services across the border	*"I took my grandchild because after visiting the hospitals here for three consecutive times and missing the vaccine, I was forced to travel to Uganda for the vaccine"*- Border Resident, Busia-Kenya.
	Formal referral by health workers at home	*"I started feeling labor pains where I visited the district hospital but got students who were attending to patients. I was bleeding and was advised to go to MASAFU (Uganda side) for delivery"*- Border resident, Busia-Kenya.
	Informal referrals by self, friends and other community members	*"…when I was sick, my employer she asked if I could have caught HIV. Therefore, she sent me to MAYENGO (Uganda side) for diagnosis, and I found that I had HIV"*-Border resident, Busia-Kenya.
*Acceptability of health services	Confidentiality of care sought across the border	*"When I discovered I was infected with HIV I was stigmatized and couldn't stand going to pick drugs as I could meet very many people I know. I was forced to go to Uganda and gained courage when picking the drugs"*-Border resident, Busia-Kenya.
	Perceived better quality of care across the border	*"When I went to the Kenyan hospital they worked on me well and I came back safely. The good thing in Kenya is that any nurse can help you, she tells you where to start from, and she directs where to get the book and notes all things concerning the baby."*- Border resident, Busia-Uganda
Experiences on how border resident communities navigated barriers during cross border health care access		
Theme	Sub themes	Illustrative quote
****Ability to physically cross into the neighbouring country	Presence of informal routes	*"Border officers send you back because all those services are available here….(in Rwanda). Actually, most people who need those services use shortcuts to cross. Furthermore, Ugandans who take HIV treatment here (in Rwanda) also pass through there (the informal route)"* – Border resident, Gatuna-Rwanda
	Official travel documents	*"No, whenever I visit there I am never asked for any document either when going or coming back"*- Border Resident, Busia-Kenya.
	Similarity in language	*"I was just treated well…It's only the language barrier that made the whole process look tiresome"*-Border Resident, Isebania-Kenya.

For peer review only

****Ability to afford care across the border	Ability to meet direct cost of care	*"The cost was so much reasonable as compared to Kenyans side and we also used Kenyan currency which is stronger that the Uganda shillings"*- Border Resident, Busia-Kenya,
	The indirect costs of care	*"While on the way we encountered the police whom we bribed to proceed with our journey to Dabani Hospital. At the hospital we had a midwife who was attending to her by bringing her food and water to bath and we ended up paying her (the midwife)"-Border Resident, Busia-Kenya.*

For peer review only

Experiences of seeking health care across the border: Lessons to inform upstream policies and system developments on cross border health in East Africa

Standards for Reporting Qualitative Research (SRQR) checklist for reporting of qualitative research

No	Topic		Page Number and line in **clean copy**
Title and abstract			
S1	Title	Concise description of the nature and topic of the study identifying the study as qualitative or indicating the approach (e.g. ethnography, grounded theory) or data collection methods (e.g. interview, focus group) is recommended	Page 1<br>Line 1-2
S2	Abstract	Summary of the key elements of the study using the abstract format of the intended publication; typically includes background, purpose, methods, results and conclusions	Page 1-2<br>Line 21-47
Introduction			
S3	Problem formulation	Description and signifcance of the problem / phenomenon studied: review of relevant theory and empirical work; problem statement	Page 4-5<br>Line 95-116
S4	Purpose or research question	Purpose of the study and specific objectives or questions	Page 5<br>Line 116-121
	Qualitative approach and research paradigm	Qualitative approach (e.g. ethnography, grounded theory, case study, phenomenolgy, narrative research) and guiding theory if appropriate; identifying the research paradigm (e.g. postpositivist, constructivist / interpretivist) is also recommended; rationale. The rationale should briefly discuss the justification for choosing that theory, approach, method or technique rather than other options available; the assumptions and limitations implicit in those choices and how those choices influence study conclusions and transferability. As appropriate the rationale for several items might be discussed together.	Page 5-6<br>Line 147-150<br>Line 175-176
	Researcher characteristics and reflexivity	Researchers' characteristics that may influence the research, including personal attributes, qualifications / experience, relationship with participants, assumptions and / or presuppositions; potential or actual interaction between researchers' characteristics and the research questions, approach, methods, results and / or transferability	Page 8<br>Line 232-235
	Context	Setting / site and salient contextual factors; rationale	Page 5<br>Line 124-140
	Sampling strategy	How and why research participants, documents, or events were selected; criteria for deciding when no further sampling was necessary (e.g. sampling saturation); rationale	Page 6<br>Line 151-160

For peer review only - http://bmjopen.bmj.com/site/about/guidelines.xhtml

	Ethical issues pertaining to human subjects	Documentation of approval by an appropriate ethics review board and participant consent, or explanation for lack thereof; other confidentiality and data security issues	Page 8<br>Line 236-241
	Data collection methods	Types of data collected; details of data collection procedures including (as appropriate) start and stop dates of data collection and analysis, iterative process, triangulation of sources / methods, and modification of procedures in response to evolving study findings; rationale	Page 6-7<br>Line 169-206
	Data collection instruments and technologies	Description of instruments (e.g. interview guides, questionnaires) and devices (e.g. audio recorders) used for data collection; if / how the instruments(s) changed over the course of the study	Page 6-7<br>Line 169-206
	Units of study	Number and relevant characteristics of participants, documents, or events included in the study; level of participation (could be reported in results)	Page 5-6<br>Line 141-150
	Data processing	Methods for processing data prior to and during analysis, including transcription, data entry, data management and security, verification of data integrity, data coding, and anonymisation / deidentification of excerpts	Page 8<br>Line 222-226
	Data analysis	Process by which inferences, themes, etc. were identified and developed, including the researchers involved in data analysis; usually references a specific paradigm or approach; rationale	Page 8<br>Line 226-235
	Techniques to enhance trustworthiness	Techniques to enhance trustworthiness and credibility of data analysis (e.g. member checking, audit trail, triangulation); rationale	Page 8<br>Line 230-235
	Syntheses and interpretation	Main findings (e.g. interpretations, inferences, and themes); might include development of a theory or model, or integration with prior research or theory	Page 11-14<br>Line 308-427
	Links to empirical data	Evidence (e.g. quotes, field notes, text excerpts, photographs) to substantiate analytic findings	Page 11-14<br>Line 308-427
	Intergration with prior work, implications, transferability and contribution(s) to the field	Short summary of main findings; explanation of how findings and conclusions connect to, support, elaborate on, or challenge conclusions of earlier scholarship; discussion of scope of application / generalizability; identification of unique contributions(s) to scholarship in a discipline or field	Page 14-17<br>Line 429-518
	Limitations	Trustworthiness and limitations of findings	Page 17<br>Line 520-525
	Conflicts of interest	Potential sources of influence of perceived influence on study conduct and conclusions; how these were managed	Page 18<br>Line 568
	Funding	Sources of funding and other support; role of funders in data collection, interpretation and reporting	Page18<br>Line 570

For peer review only
